# Epigenetically silenced lncRNA SNAI3-AS1 promotes ferroptosis in glioma via perturbing the m^6^A-dependent recognition of Nrf2 mRNA mediated by SND1

**DOI:** 10.1186/s13046-023-02684-3

**Published:** 2023-05-19

**Authors:** Jianglin Zheng, Qing Zhang, Zhen Zhao, Yue Qiu, Yujie Zhou, Zhipeng Wu, Cheng Jiang, Xuan Wang, Xiaobing Jiang

**Affiliations:** 1 Department of Neurosurgery, Union Hospital, Tongji Medical College, Huazhong University of Science and Technology, Wuhan, Hubei China; 2 Department of Otolaryngology, Union Hospital, Tongji Medical College, Huazhong University of Science and Technology, Wuhan, Hubei China; 3 Department of Neurosurgery, Weifang People’s Hospital, Weifang, Shandong China

**Keywords:** Glioma, LncRNA, Ferroptosis, m^6^A, Nrf2

## Abstract

**Background:**

Ferroptosis has been linked to tumor progression and resistance to antineoplastic therapy. Long noncoding RNA (lncRNA) exerts a regulatory role in various biological processes of tumor cells, while the function and molecular mechanism of lncRNA in ferroptosis are yet to be clarified in glioma.

**Methods:**

Both gain-of-function and loss-of-function experiments were employed to investigate the effects of SNAI3-AS1 on the tumorigenesis and ferroptosis susceptibility of glioma in vitro and in vivo. Bioinformatics analysis, Bisulfite sequencing PCR, RNA pull-down, RIP, MeRIP and dual-luciferase reporter assay were performed to explore the low expression mechanism of SNAI3-AS1 and the downstream mechanism of SNAI3-AS1 in ferroptosis susceptibility of glioma.

**Results:**

We found that ferroptosis inducer erastin downregulates SNAI3-AS1 expression in glioma by increasing the DNA methylation level of SNAI3-AS1 promoter. SNAI3-AS1 functions as a tumor suppressor in glioma. Importantly, SNAI3-AS1 enhances the anti-tumor activity of erastin by promoting ferroptosis both in vitro and in vivo. Mechanistically, SNAI3-AS1 competitively binds to SND1 and perturbs the m^6^A-dependent recognition of Nrf2 mRNA 3’UTR by SND1, thereby reducing the mRNA stability of Nrf2. Rescue experiments confirmed that SND1 overexpression and silence can rescue the gain- and loss-of-function ferroptotic phenotypes of SNAI3-AS1, respectively.

**Conclusions:**

Our findings elucidate the effect and detailed mechanism of SNAI3-AS1/SND1/Nrf2 signalling axis in ferroptosis, and provide a theoretical support for inducing ferroptosis to improve glioma treatment.

**Supplementary Information:**

The online version contains supplementary material available at 10.1186/s13046-023-02684-3.

## Background

Glioma is the most frequent primary malignancy in central nervous system, characterized by high aggressiveness and high recurrence rate [1]. Despite extensive efforts to develop available therapeutic options, the prognosis of patients with glioma remains unsatisfactory, especially for glioblastoma. Resistance to cell death is a vital distinctive hallmark of tumor [2], which favors tumor progression and inevitably leads to intrinsic or acquired treatment resistance for most patients with glioma. Hence, exploring the mechanisms of resistance to cell death is essential for improving the outcome of patients with glioma.

Ferroptosis, a newly identified type of cell death, is distinct from other types of cell death in its unique genetic, metabolic, biochemical, and morphological features [3]. The accumulation of iron-dependent lipid peroxidation is the direct driver of ferroptotic cell death [4]. Altered iron homeostasis, abnormal glutathione metabolism, and dysregulated lipid metabolism converge to control the initiation and execution of ferroptosis. Mitochondrial abnormalities were dominant morphological features of cells undergoing ferroptosis, including shrunken mitochondria, increased membrane density and reduced mitochondrial ridges [4]. Recent findings have linked ferroptosis to oncological research, including tumor progression and resistance to antineoplastic therapy. For example, the circular RNA circLRFN5 can inhibit the progression of glioblastoma by inducing ferroptosis [5]. The ferroptosis inducer erastin exerts temozolomide-sensitizing effect on glioma cells [6]. Nevertheless, the genetic and mechanistic details underlying ferroptosis in glioma remain obscure. Further studies in this context are needed.

The long non-coding RNA (lncRNA), defined as a particular class of non-coding RNAs with a length of > 200 nucleotides, has been reported to participate in the regulation of tumor cell resisting death, including ferroptosis [7]. For instance, lncRNA P53RRA promotes ferroptosis in lung cancer by nuclear sequestration of p53 [8]. lncRNA MT1DP can sensitizes erastin-induced ferroptosis in non-small cell lung cancer via miR-365a-3p/Nrf2 axis [9]. Our previous study constructed a prognostic ferroptosis-related lncRNAs signature in glioma, and screened out 8 lncRNAs with differential expression levels between glioma tissues and non-tumor brain tissues [10]. However, the specific biological roles and related mechanisms of these lncRNAs in glioma progression and ferroptosis remain unclear, which is also an issue that we sought to address.

In this study, we focused on lncRNA SNAI3-AS1, whose expression in glioma cells were suppressed by erastin due to increasing DNA methylation level within its promoter. We found that SNAI3-AS1 inhibits glioma cell proliferation, migration, and invasion. We also confirmed that SNAI3-AS1 perturbs the m^6^A recognition of Nrf2 mRNA 3’UTR by SND1 to decrease Nrf2 mRNA stability, thereby promoting erastin-induced ferroptosis in glioma. Our study elucidates the effect and detailed mechanism of SNAI3-AS1/SND1/Nrf2 in ferroptosis, and provides a theoretical support for inducing ferroptosis to improve glioma treatment.

## Methods

### Patients and tissue specimens

A total of 24 glioma samples together with corresponding non-neoplastic brain tissues were obtained from patients who underwent operative management at the Neurosurgery Department of Wuhan Union Hospital between May 2020 to Jan 2022. Detailed information of patients was provided in Supplementary Table 1. None of the patients had received anti-cancer therapy prior to surgery. All specimens were excised and immediately snap frozen in liquid nitrogen. All study protocols were approved by the institutional review board of Union Hospital, Tongji Medical College, Huazhong University of Science and Technology (No. 0608), and written informed consents were obtained from all participants.

### Cell lines, reagents, plasmids, and siRNAs

The glioma cell lines (U87MG, U251, A172, LN229, U373, and T98G) and human kidney epithelial cell line 293 T were purchased from the American Type Culture Collection (ATCC). The normal astrocyte cell line (HA1800) was obtained from Science Cell Laboratory. All cell lines were maintained in the incubator at 37 °C with a 5% CO2 humidified atmosphere and cultured in DMEM with 10% fetal bovine serum (FBS, Gibco, USA) and 10 mg/mL penicillin and streptomycin (Beyotime, China). Cells were used within 6 months of culturing and regularly tested for Mycoplasma contamination to ensure mycoplasma-free.

5-Azacytidine (HY-10586), Erastin (HY-15763), Ferrostatin-1 (HY-100579), Deferoxamine (HY-B1625), and cycloheximide (CHX, HY-12320) were purchased from MedChemExpress (MCE, Shanghai, China). The lentiviral overexpression plasmids of SNAI3-AS1 and SND1, and the lentiviral knockdown plasmids of SNAI3-AS1 were synthesized by Genomeditech (Shanghai, China). The sequences of used shRNAs and siRNAs were listed in Supplementary Table 2. Human SND1 (NM_014390.4) and corresponding truncated structure cDNAs were amplified by PCR and cloned into pECMV-3xFLAG-C expression vector. Lipofectamine 3000 (Thermo Fisher Scientific, USA) was used for transient transfections according to manufacturers protocol, and cells stably expressing colonies were selected with 2 mg/ml puromycin.

### Nuclear and cytoplasmic extraction, RNA extraction, and RT-qPCR

Nuclear and cytoplasmic RNAs were separated using a nuclear and cytoplasmic RNA purification kit (AM1921, Thermo Fisher Scientific). Total RNA from tissues or cell lines were extracted using Trizol (Takara, Otsu, Japan) based on the manufacturer’s instructions and assessed via Nanodrop. By using Evo M-MLV RT Kit (AG11728, Accurate Biotechnology Co., Ltd, Hunan, China), 1 ug of total RNA was reverse transcribed into cDNA, which was used for subsequent RT-qPCR via SYBR Green premix Pro Taq HS qPCR Kit (AG11728, Accurate Biotechnology Co., Ltd). Each reaction was performed on the Bio-Rad Real time PCR system (Bio-Rad, USA). The internal reference for normalization of RT-qPCR results was GAPDH, and relative expression was calculated using the 2(-ΔΔCT) method. The primers involved were listed in Supplementary Table 3.

### Bisulfite sequencing PCR (BSP)

Genomic DNA from glioma cells under different treatments was extracted using a Genomic DNA Kit (G3633, Servicebio, China). Specific BSP primers were designed based on the sequence of transcription start site of SNAI3-AS1 (Supplementary Table 3). The next steps in turn were bisulfite treatment of genomic DNA, PCR amplification, electrophoresis, DNA purification, transformation, PCR amplification identification, and DNA sequencing. Servicebio provided specific technical support.

### Western blotting and antibodies

Cells were lysed in RIPA buffer (P0013B, Beyotime) with protease inhibitors (GRF101, Epizyme, China). After centrifugation at 14000 × g, 4 °C for 15 min, proteins were denatured using 5 × loading buffer (G2013-1ML, Servicebio) and boiled for 10 min. Equal quantities of total protein per lane were separated by SDS-PAGE and transferred to PVDF membranes (IPVH00010, Merck Millipore). The membranes were washed in 5% non-fat dry milk in TBST for 1 h, and were then incubated at 4 °C overnight with a different primary antibody. Next day, membranes were incubated with the corresponding secondary antibodies for 1 h at room temperature, and visualized with Enhanced Chemiluminescent (ECL, New Cell & Molecular Biotech, China) by using ChemiDoc Imaging Systems (Bio-Rad).

The primary antibodies were listed as following: GAPDH (60,004–1-Ig, Proteintech), β-actin (66009–1-Ig, Proteintech), Nrf2 (16396–1-AP, Proteintech), SND1 (10760–1-AP, Proteintech), METTL3 (15073–1-AP, Proteintech), METTL14 (26158–1-AP, Proteintech), WTAP (10200–1-AP, Proteintech), FLAG (20543–1-AP, Proteintech), Keap1 (10503–2-AP, Proteintech), GPX4 (67763–1-Ig, Proteintech), 4-Hydroxynonenal (4-HNE, R&D systems, MAB3249). Secondary antibodies used were HRP-conjugated Affinipure Goat Anti-Mouse IgG (H + L) (SA00001-1, Proteintech) and HRP-conjugated Affinipure Goat Anti-Rabbit IgG (H + L) (SA00001-2, Proteintech).

### RNA pull-down and RIP assays

Full-length or truncated SNAI3-AS1 specific probes and negative control (NC) probe were synthesized from GeneCreate Biological Engineering (Wuhan, China). RNA pull-down assay was conducted by using a Pierce Magnetic RNA–Protein Pull-Down Kit (Thermo Fisher Scientific) according to the manufacturer’s instructions. The co-precipitated proteins eluted from the beads were separated by SDS-PAGE and then silver stained by using a Fast Silver Stain Kit (P0017S, Beyotime) following the manufacturer’s recommendations. Mass spectrometry was carried out by SpecAlly Life Technology Co., Ltd (Wuhan, China).

For RIP assay, the Magna RIP RNA-Binding Protein Immunoprecipitation Kit (Millipore, Bedford, MA, USA) was used according to the manufacturer’s instructions.

Cell lysates obtained from 3–4 × 10^7^ cells were subjected to immunoprecipitation at 4 °C overnight with primary antibodies against SND1 (10760–1-AP, Proteintech) or FLAG (20543–1-AP, Proteintech). The isotype control was a homologous IgG. TRIzol reagent was used for extracting RNA samples, which were then tested through RT-qPCR.

### Fluorescent in situ hybridization (FISH) and Immunofluorescence

A Cy3-labeled SNAI3-AS1 FISH probe was purchased from RiboBio (Guangzhou, China) and FISH assays were carried out with a Ribo FISH kit (C10910, RiboBio) according to the manufacturer’s instructions. For immunofluorescence staining assays, cells were fixed with 4% paraformaldehyde at room temperature for 15 min, permeated with 0.5% Triton X-100 for 10 min, blocked with 5% BSA for 1 h, incubated with primary anti-bodies at 4 ◦C overnight and then with corresponding Fluor-labeled secondary antibodies (1:200, Thermo Fisher Scientific). Nuclei was stained via DAPI (C1002, Beyotime) was used to stain nuclei. Images were taken by fluorescence microscope (Nexcope NE930, Ningbo, China).

### Immunohistochemistry (IHC) assay and Terminal deoxynucleotidyl transferase dUTP nick end labeling (TUNEL) assay

Tissue specimens from human or mouse were fixed with 4% paraformaldehyde, embedded in paraffin, sectioned with 6-μm thickness, and immunostained with specific antibodies, including SND1 (10,760–1-AP, Proteintech), Nrf2 (16,396–1-AP, Proteintech), GPX4 (67,763–1-Ig, Proteintech), and 4-HNE (R&D systems, MAB3249). The histological slides were observed under a light microscope (Leica, Germany). The percentage of positive cells was calculated.

TUNEL staining was performed with In Situ Cell Death Detection Kit, POD (Roche, Switzerland) according to the manufacturer’s protocol. Images were acquired with an Olympus FSX100 microscope (Olympus, Tokyo, Japan).

### m.^6^A dot-blot assay and methylated RNA immunoprecipitation (MeRIP)

The m^6^A dot-blot assay was conducted to assess the total RNA m^6^A levels. In brief, total RNA was extracted using Trizol. After denatured with 95℃ for 3 min and chilling on ice, mRNA was spotted on Biodyne Nylon Transfer Membranes (Pall) and cross-linked for 30 s by UVP (1.2 kJ). The anti-m^6^A antibody (ab151230, Abcam) was applied to determine the global m^6^A level.

Methylated RNA immunoprecipitation (MeRIP) assay was performed by using the Magna MeRIP m6A Kit (Millipore, USA) to quantify the m^6^A modification of RNA samples. Briefly, after the removal of genomic DNA via DNase, total RNA was subjected to fragmentation. Magnetic beads were incubated with 10 μg anti-m^6^A antibody or IgG, followed by immunoprecipitation reaction with RNA samples. Afterwards, the m^6^A-enriched RNA fragments were eluted via N^6^-methyladenosine 5’-monophosphate salt, reverse transcribed, and quantified by RT-qPCR. According to the sequences with predicted m^6^A sites, primers were designed for MeRIP-qPCR analysis (Supplementary Table 3). Relative m^6^A enrichment was normalized to the input.

### Dual-luciferase reporter assays and mRNA stability assays

By using psi-CHECK-2 vector, a total of four dual-luciferase reporters, including SNAI3-AS1 promoter, Nrf2 promoter, Nrf2 mRNA 3’UTR_WT, and Nrf2 mRNA 3’UTR_Mut, were synthesized by GeneCreate Biological Engineering. Detailed sequences were listed in Supplementary Table 4–6. In brief, cells seeded in 24-well plates with 60–80% confluency were transfected with dual-luciferase reporters. Forty-eight hours after transfection, renilla and firefly luciferase activities in each well were detected by using HBLumi Dual-luciferase reporter assay kit (Hanbio, Shanghai, China) according to the manufacturer’s instructions.

To evaluate the RNA stability, cells were treated by actinomycin D (5 μg/ml, M4881, AbMole BioScience) for 0, 2, 4, 6 h. The total RNA was extracted by Trizol Reagent and analyzed by RT-qPCR as described above. The results were normalized to the values measured at 0 h.

### Cell viability, colony formation, and EdU assays

The Cell viability was measured by the Cell Counting Kit-8 (CCK8, BS350, Biosharp, Hefei, China). Cell suspension was planted into 96-well plates (3 × 10^3^ cells/well) with 100 μl of medium. Subsequently, 10 μl CCK8 solution was added to each well at the time points of 0, 24, 48, 72, and 96 h. After incubation of 2 h at 37℃ absorbance at 450 nm of each well was measured.

For Colony formation assay, glioma cells were cultured in 6-well plates with 1 × 10^3^ cells per well. After 2 weeks of incubation, the cells were washed thrice with PBS, fixed with 4% paraformaldehyde for 15 min, and stained with 0.1% crystal violet solution for 12 min. The size and number of colonies were observed.

For EdU assays, glioma cells were cultured in 48-well plates until 50%-80% confluent. Then, BeyoClick™ EdU Cell Proliferation Kit with Alexa Fluor 594 (C0078L, Beyotime) was used according to the manufacturer’s instructions. The proportion of EdU-positive cells were quantified under a fluorescence microscope (Nexcope NE930, Ningbo, China).

### Cell migration, invasion, and cycle assays

The cell migration and invasion assays were performed using transwell chambers with or without Matrigel membranes. In brief, a total of 1 × 10^4^ starved glioma cells were added to the upper chamber with 200 μl of serum-free medium, and the lower chamber was applied with 600 μL of complete medium containing 20% FBS. After incubation for 36 h, cells attached to the upper surface were removed with a cotton, while cells that migrated or invaded into the lower surface of the membrane were fixed with 4% paraformaldehyde for 15 min, and stained with 0.1% crystal violet solution for 12 min, and counted in five random fields of view under an objective lens.

For cell cycle assays, cells were collected and fixed in 75% ice-cold ethanol at 4 °C overnight. Next day, the fixed cells were washed with PBS thrice and stained with propidium iodide (Beyotime). Lastly, cell cycle analysis was performed using flow cytometry, and the results were visualized via ModFit LT software.

### Malondialdehyde (MDA), iron, and lipid ROS assays

The Lipid Peroxidation MDA Assay Kit (S0131M, Beyotime) was used to determine the relative MDA concentration in cell lysate according to the manufacturer’s instructions. In short, glioma cells in 10-cm dishes with the treatment of erastin or DMSO for 48 h were lysed via western blotting and immunoprecipitation (IP) (P0013, Beyotime). Then, cell homogenates were centrifugated at 13,000 g for 10 min, and 100 μl of obtained supernatant was mixed with 200 μl of MDA working solution and incubated at 100 ◦C for 15 min. After cooling to room temperature, the absorbance of each mixture was measured at 532 nm.

Intracellular ferrous iron (Fe^2+^) level was measured by using the iron assay kit (ab83366, Abcam) according to the manufacturer’s instructions. Briefly, glioma cells were seeded in a 10-cm plate and treated with erastin or DMSO for 48 h. Then, Cells were harvested, washed in ice cold PBS, and homogenized in 5 × volumes of iron assay buffer on ice. The supernatant was obtained after centrifugation (13,000 × g, 10 min) at 4 °C, mixed with iron reducer, and incubated at room temperature for 30 min. Subsequently, each sample mixed with 100 μl of iron probe was incubated at room temperature in the dark for 1 h. The absorbance at 593 nm was measured immediately using a colorimetric microplate reader.

Lipid ROS level was detected by flow cytometry via BODIPY-C11 dye (D3861, Thermo Fisher Scientific). Glioma cells were seeded in 6-well plates and treated with erastin or DMSO for 48 h. After washing with PBS, cells were stained with 2 ml complete medium containing 5 µM of BODIPY-C11 dye and incubated at 37 °C for 30 min in the dark. Then, cells were washed thrice with PBS to remove excess labeling mixture followed by resuspending in 200 μl medium. Oxidation of BODIPY-C11 resulted in a shift of the fluorescence emission peak from 590 to 510 nm proportional to lipid ROS generation. More than 10,000 cells were analyzed by flow cytometric to determine the intracellular lipid ROS level per condition. Data were processed using the FlowJo Software.

### Transmission electron microscopy

In brief, glioma cells were seeded into T25 culture flask and were treated with Erastin or DMSO for 48 h. Then, Cells are collected after centrifuge and the precipitation. The IEM fixative was added to cells and let the cell precipitation resuspended in the fixative, and then fixed at 4 °C for preservation. Images were obtained through transmission electron microscope (Hitachi, HT7700, Japan).

### Xenograft mouse models

All animal experiments were approved by the Institutional Animal Care and Use Committee of Huazhong University of Science and Technology ([2022] IACUC Number: 3217), and performed according to NIH animal care guidelines. Female BALB/c-nude mice aged 6 weeks (BIONT, Wuhan, China) were used to construct orthotopic glioma model. Briefly, a total of 5 × 10^5^ U87MG cells stably expressing firefly luciferase (Fluc) with indicated treatments were injected into the mouse brain at 2 mm lateral, 2 mm posterior to the bregma, and 2 mm depth via a stereotaxic apparatus. Tumor growth was monitored using bioluminescence imaging (Bruker Corporation, Billerica, MA). The mouse weight and survival time were carefully recorded. Tumor tissues harvested from mice were used to perform corresponding staining.

### Bioinformatics analysis

The transcript data of glioma samples together with clinical information was downloaded from The Cancer Genome database (TCGA, https://xenabrowser.net/heatmap/) and the Chinese Glioma Genome Atlas mRNAseq_693 database (CGGA693, http://www.cgga.org.cn/index.jsp). The transcript data of normal brain tissues was downloaded from the Genotype-Tissue Expression (GTEx; https://gtexportal.org/home/) database. The DNA methylation status of SNAI3-AS1 were downloaded from TCGA. The CpG island methylation analysis of SNAI3-AS1 was performed via MethPrimer (http://www.urogene.org/cgi-bin/methprimer/methprimer.cgi). Ferroptosis-related genes were obtained from FerrDb V2 (http://www.zhounan.org/ferrdb/current/).

### Statistical analyses

Graphpad prism 8.0 was applied to perform statistical analyses. The data were presented as mean ± standard deviation. Spearman rank correlation was used for correlation analysis. Statistical comparisons between two groups were carried out by Student’s t-test. Mouse survival data was analyzed by the Kaplan–Meier log-rank test. *P* < 0.05 was considered to reflect a statistically significant difference. All experiments were repeated at least three times.

## Results

### Ferroptosis inducer erastin inhibits SNAI3-AS1 expression by increasing DNA methylation level at the CpG islands within its promoter

**Fig. 1:**
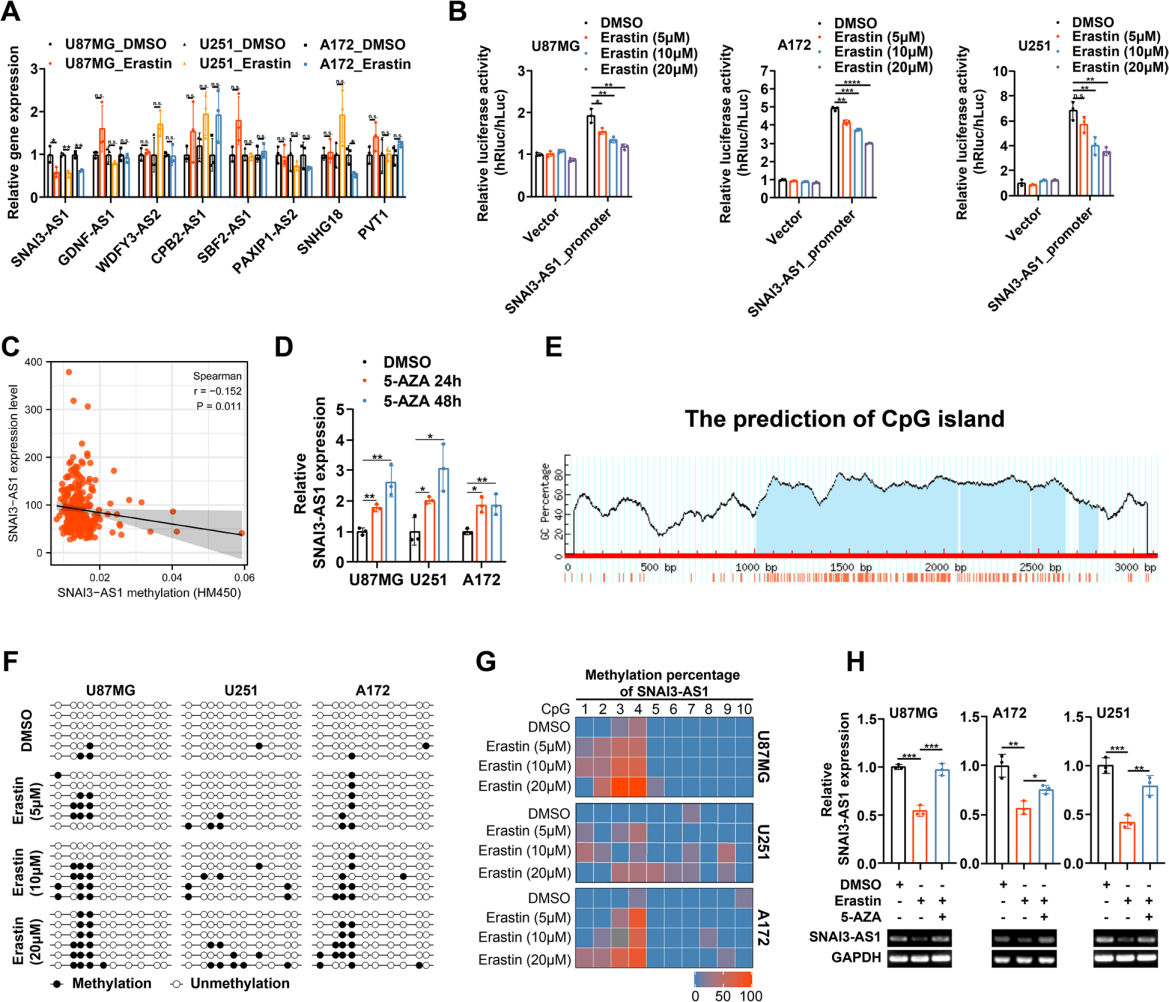
Ferroptosis inducer erastin downregulates SNAI3-AS1 expression by increasing DNA methylation level of its promoter. **A** The expression of eight candidate lncRNAs in U87MG, U251 and A172 cells under erastin (10 μM, 48 h) treatments as measured by RT-qPCR. **B** Dual-luciferase reporter assays showed the transcription activity of SNAI3-AS1 promoter after erastin (5/10/20 μM, 48 h) treatments. **C** The correlation between DNA methylation level and gene expression of SNAI3-AS1 according to cBioPortal database. **D** The expression of SNAI3-AS1 in U87MG, U251 and A172 cells under 5-AZA (1 μM, 24/48 h) treatments as measured by RT-qPCR. **E** A Prediction analysis of CpG islands in the sequence range of 3100 bp upstream from the transcriptional start site in the SNAI3-AS1 promoter region. **F** BSP results of SNAI3-AS1 methylation status in U87MG, U251 and A172 cells under erastin (5/10/20 μM, 48 h) treatments. **G** Heat map of methylation percentage of SNAI3-AS1 in U87MG, U251 and A172 cells under erastin (5/10/20 μM, 48 h) treatments. **H** The expression of SNAI3-AS1 in U87MG, U251 and A172 cells under erastin (10 μM, 48 h) or erastin (10 μM, 48 h) combined with 5-AZA (1 μM, 48 h) treatments as measured by RT-qPCR and agarose gel image. *P < 0.05, **P < 0.01, ***P < 0.001, and n.s., not significant

We previously constructed a prognostic ferroptosis-related lncRNAs signature, and screened out 8 lncRNAs with differential expression levels between glioma tissues and non-tumor brain tissues [10]. To explore the specific role and mechanism of these involved lncRNAs in glioma progression and ferroptosis, three glioma cell lines, U87MG, U251 and A172, were treated with ferroptosis inducer erastin for transcriptome analysis with RT-qPCR. Following erastin treatments with different concentration, only SNAI3-AS1 showed significant changes with the same down-regulated trends in all three glioma cell lines (Fig. 1A and Supplementary Fig. 1). Thus, we focus on SNAI3-AS1 for further investigation.

Firstly, we attempted to elucidate the mechanism of SNAI3-AS1 inhibited by erastin. Dual-luciferase reporter plasmid containing the promoter fragment of SNAI3-AS1 was constructed to examine whether SNAI3-AS1 attenuation occurred at the transcriptional or post-transcriptional levels. Dual-luciferase reporter assays showed that relative luciferase activities were markedly reduced by erastin treatment in a concentration-dependent manner, which indicated that erastin-induced downregulation of SNAI3-AS1 occurred at the transcriptional level (Fig. 1B). It is well-known that DNA methylation is one of the most common epigenetic modifications, and referred as a repressive mark of gene repression [11]. Next, we sought to analyze the relationship between DNA methylation level and gene expression of SNAI3-AS1. We observed that SNAI3-AS1 expression was negatively linked with its DNA methylation status from cBioPortal database (Fig. 1C). In the glioma cohort from TCGA, there were 4 CpG sites whose methylation levels were negatively correlated with SNAI3-AS1 expression (Supplementary Fig. 2A-R). After treatment with DNA demethylating drug 5-AZA, glioma cells exhibited elevated SNAI3-AS1 expression. These results indicated that increased DNA methylation level can represses SNAI3-AS1 expression (Fig. 1D). Then, we performed Bisulfite sequencing PCR (BSP) on U87MG, U251 and A172 cells under different treatment conditions to assess the impact of erastin to SNAI3-AS1 DNA methylation (Fig. 1E and Supplementary Fig. 2S). As expected, erastin treatment increased the DNA methylation level of SNAI3-AS1 in a concentration-dependent manner (Fig. 1F, G). Additionally, 5-AZA could rescue the erastin-induced downregulation of SNAI3-AS1 (Fig. 1H). Together, ferroptosis inducer erastin inhibits SNAI3-AS1 expression by increasing DNA methylation level at the CpG islands within its promoter.

### SNAI3-AS1 functions as a tumor suppressor in glioma

To uncover the physiological role of SNAI3-AS1 in glioma progression, we first investigated the expression of SNAI3-AS1 in glioma tissues and normal tissues. By surveying TCGA and CGGA693 databases, we observed that SNAI3-AS1 expression was obviously downregulated in glioma, and was inversely correlated with WHO grade (Supplementary Fig. 3A, B). To validate the results of public databases, 24 paired glioma tissues and corresponding nontumoral brain tissues were collected, and RT-qPCR showed that SNAI3-AS1 expression was reduced in glioma tissues (Supplementary Fig. 3C). In addition, we found that SNAI3-AS1 expression was markedly downregulated in several glioma cell lines compared with that of normal astrocyte (Supplementary Fig. 3D). Furthermore, Kaplan–Meier analysis demonstrated that glioma patients with lower SNAI3-AS1 expression had a worse overall survival (Supplementary Fig. 3E, F). The receiver operating characteristics (ROC) curve also confirmed SNAI3-AS1 performed satisfactorily in prognostic analysis of glioma (Supplementary Fig. 3G, H). Overall, lncRNA SNAI3-AS1 is downregulated in glioma and can serve as a prognostic biomarker.

**Fig. 2:**
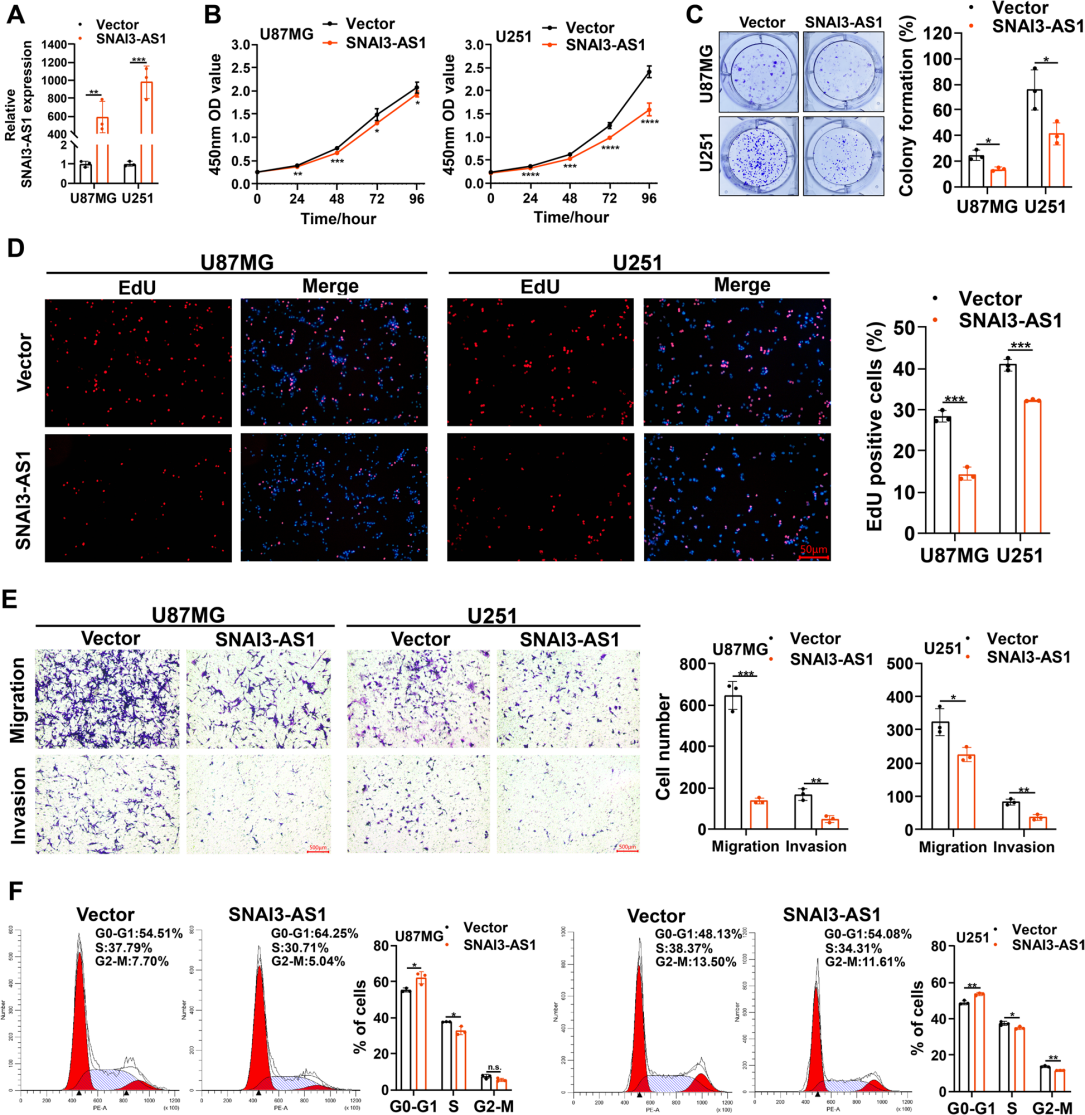
SNAI3-AS1 inhibits the proliferation, invasion, and migration of glioma cells in vitro. **A** RT-qPCR was used to detect the expression of SNAI3-AS1in U87MG and U251 cells transfected with SNAI3-AS1 overexpressed lentiviral vector or control vector. **B** The growth curves of transfected U87MG and U251 cells were determined by CCK8 assays. **C** The colony formation assays were performed in transfected U87MG and U251 cells. **D** The proliferation of transfected U87MG and U251 cells was detected by EdU staining assays. **E** The transwell assays showed the migration and invasion abilities of transfected U87MG and U251 cells. **F** Cell cycle distributions of transfected U87MG and U251 cells were measured by flow cytometry. *P < 0.05, **P < 0.01, ***P < 0.001, and n.s., not significant

Next, we constructed one SNAI3-AS1 overexpressed lentiviral vector and two SNAI3-AS1 specific shRNAs. Given that the SNAI3-AS1 expression was relative lower in U87MG and U251, but higher in A172, we generated U87MG and U251 cell lines stably overexpressing SNAI3-AS1, and A172 cell line stably knocking down SNAI3-AS1 (Fig. 2A and Supplementary Fig. 4A). CCK8, colony formation, and EdU staining assays revealed that SNAI3-AS1 overexpression inhibited proliferative ability of glioma cells (Fig. 2B-D). Transwell assays displayed that SNAI3-AS1 overexpression significantly reduced cell migration and invasion capacities (Fig. 2E). In addition, cell cycle assays showed that SNAI3-AS1 overexpression leaded to cell cycle arrest at the G0-G1 stage, which means cell proliferation decreased (Fig. 2F). Contrary to the above results, SNAI3-AS1 knockdown significantly enhanced the proliferation, migration, and invasion abilities of A172 cells (Supplementary Fig. 4B-F). Together, these findings firstly determine the oncosuppressive role of SNAI3-AS1 in glioma growth and aggressiveness.

### SNAI3-AS1 promotes erastin-induced ferroptosis in vitro

**Fig. 3:**
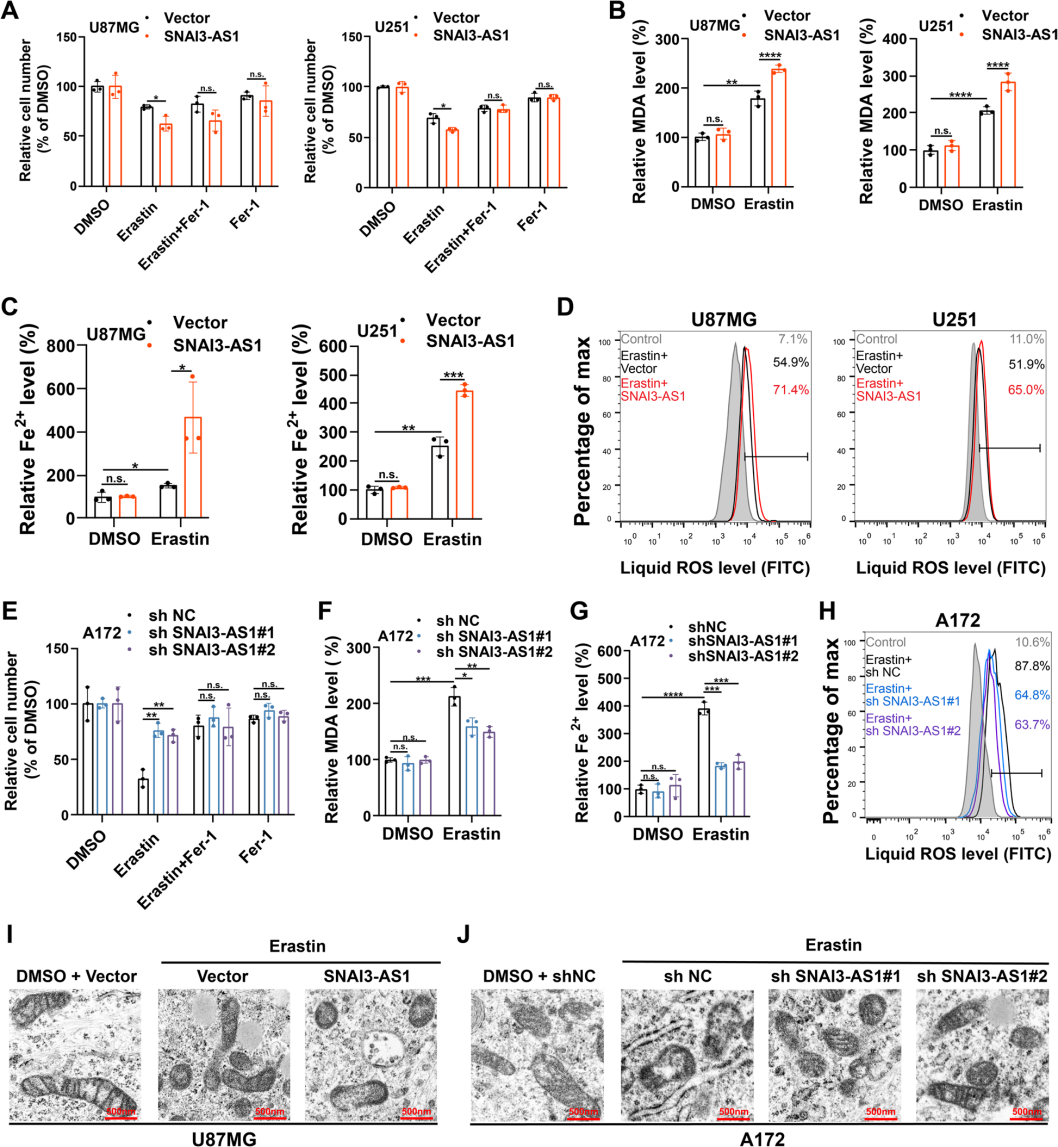
SNAI3-AS1 promotes erastin-induced ferroptosis in vitro. **A-D** U87MG and U251 cells stably overexpressing SNAI3-AS1 were treated with erastin (10 μM) ± ferrostatin-1 (2 μM) for 48 h, cell viabilities were detected via CCK8 assays (**A**), intracellular MDA was determined by MDA assays (**B**), intracellular Fe^2+^ was measured by iron detection assays (**C**), lipid ROS accumulation was analyzed by flow cytometry with C11-BODIPY staining (**D**). **E–H** A172 cells with stable SNAI3-AS1 knockdown were treated with erastin (10 μM) ± ferrostatin-1 (2 μM) for 48 h, cell viabilities were detected via CCK8 assays (**E**), intracellular MDA was determined by MDA assays (**F**), intracellular Fe^2+^ was measured by iron detection assays (**G**), lipid ROS accumulation was analyzed by flow cytometry with C11-BODIPY staining (**H**). Transmission electron microscopy was performed to evaluate the ultrastructural changes of mitochondria in U87MG stably overexpressing SNAI3-AS1 (**I**) and A172 cells with stable SNAI3-AS1 knockdown (**J**) after treated with Erastin (10 μM) for 48 h. *P < 0.05, **P < 0.01, ***P < 0.001, ****P < 0.0001, and n.s., not significant

Recent studies reported that under ferroptosis-inducing conditions, tumor cells initiated coping mechanisms for stress through epigenetic alterations [12, 13]. Therefore, we speculated that SNAI3-AS1, which was inhibited by erastin, might participate in the regulation of ferroptosis. Using CCK8 assays, we observed that SNAI3-AS1 overexpression caused decreased cell viability due to increased ferroptotic events (Fig. 3A and Supplementary Fig. 5A, B). Moreover, the promotion of erastin-induced ferroptosis caused by SNAI3-AS1 overexpression was dose- and time-dependent to some extent (Supplementary Fig. 5C-F). It is well known that Fe^2+^ and lipid ROS are two key determinants of ferroptosis process, and malondialdehyde (MDA) is the end-product of lipid ROS. Next, we detected their concentrations in erastin-treated glioma cells. Results showed that SNAI3-AS1 overexpression significantly increased the accumulation of MDA, Fe^2+^, and lipid ROS (Fig. 3B-D). Whereas, we observed that knockdown of SNAI3-AS1 promoted cell viability due to reduced ferroptotic events (Fig. 3E and Supplementary Fig. 5G). Glioma cells became more resistance to erastin-induced ferroptosis in a dose- and time-dependent manner with the knockdown of SNAI3-AS1(Supplementary Fig. 5H, I). Consistently, in erastin-treated glioma cells, SNAI3-AS1 knockdown obviously reduced the intracellular levels of MDA, Fe^2+^, and lipid ROS (Fig. 3F-H). We also performed transmission electron microscopy (TEM) analysis to describe the specific morphological changes in ferroptotic cell death. As expected, the ultrastructural changes of mitochondria, such as shrunken mitochondria, increased membrane density and reduced mitochondrial ridges, were more prominent after overexpressing SNAI3-AS1, and partly recovered following knockdown of SNAI3-AS1 (Fig. 3I-J). Taken together, above results demonstrates that SNAI3-AS1 enhances the anti-tumor activity of erastin by promoting ferroptosis in vitro.

### SNAI3-AS1 inhibits mRNA stability of Nrf2

**Fig. 4:**
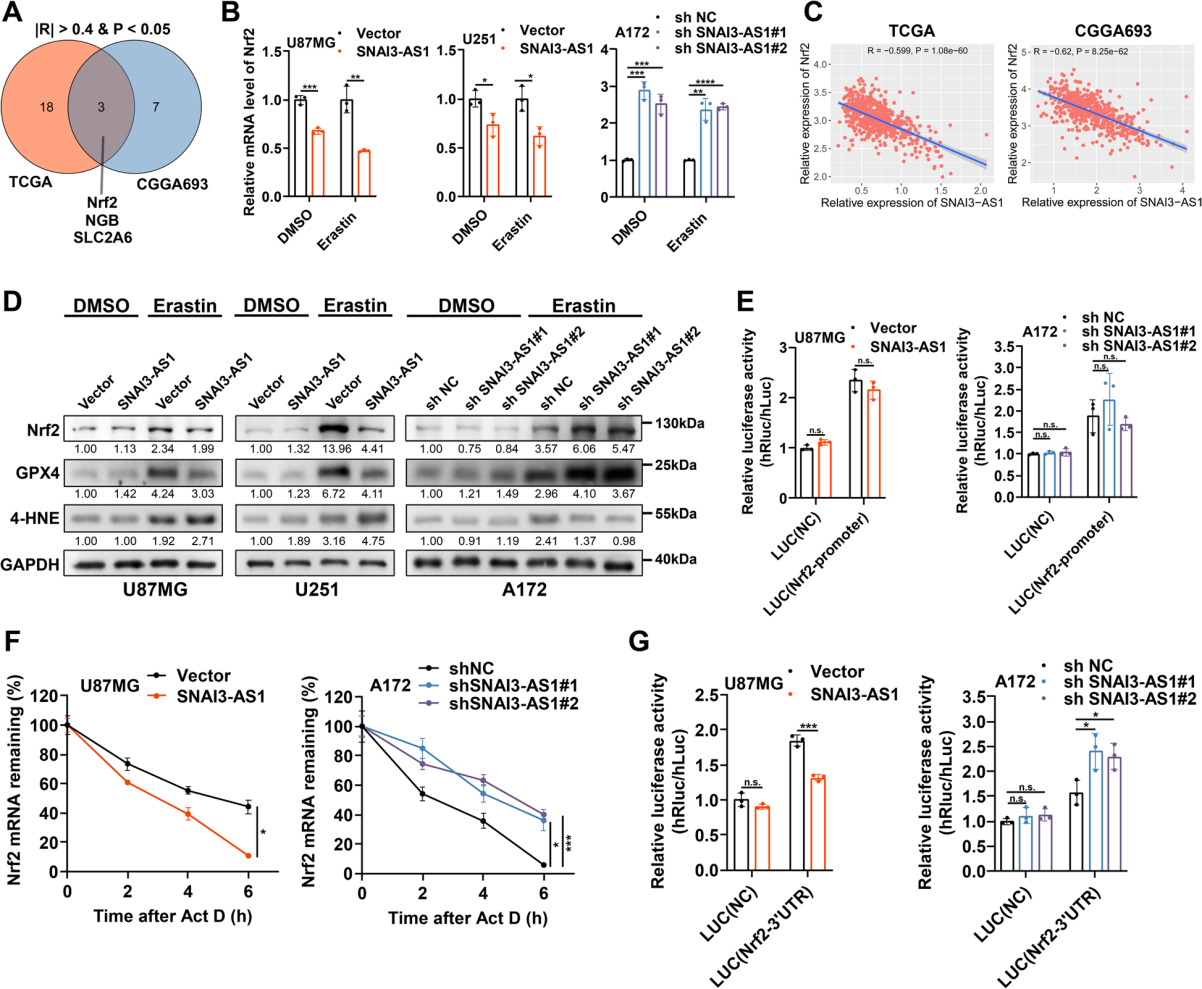
SNAI3-AS1 inhibits mRNA stability of Nrf2. **A** A Venn Diagram showed the intersection result of correlation analysis between SNAI3-AS1 and ferroptosis-related genes in TCGA and CGGA693 databases. **B** RT-qPCR was used to detect the mRNA level of Nrf2 after SNAI3-AS1 overexpression or knockdown under DMSO and erastin (10 μM, 48 h) treatments. **C** The correlation between SNAI3-AS1 and Nrf2 in TCGA and CGGA693 databases. **D** Western blotting showed the level of Nrf2, GPX4 and 4-HNE after SNAI3-AS1 overexpression or knockdown under DMSO and erastin (10 μM, 48 h) treatments. **E** U87MG cells with SNAI3-AS1 overexpression and A172 cells with SNAI3-AS1 knockdown were transfected with a dual luciferase reporter plasmid containing Nrf2 promoter. The relative luciferase activity was measured and normalized. **F** After actinomycin D (5 μg/ml) treatment for 0, 2, 4, 6 h, RT-qPCR was used to analysis the Nrf2 mRNA stability in U87MG cells with SNAI3-AS1 overexpression and A172 cells with SNAI3-AS1 knockdown. **G** U87MG cells with SNAI3-AS1 overexpression and A172 cells with SNAI3-AS1 knockdown were transfected with a dual luciferase reporter plasmid containing Nrf2 mRNA 3’UTR. The relative luciferase activity was measured and normalized. *P < 0.05, **P < 0.01, ***P < 0.001, ****P < 0.0001, and n.s., not significant

Next, we attempted to understand the mechanism by which SNAI3-AS1 confers ferroptosis susceptibility in glioma cells. Considering there is currently no study reporting the function and mechanism of SNAI3-AS1 on ferroptosis, we first investigated whether there is a correlation between SNAI3-AS1 and ferroptosis-related genes in TCGA and CGGA693 databases. After taking an intersection of the results obtained from two databases with the absolute value of spearman correlation coefficient greater than 0.4 and *P* value less than 0.05, we found that only three candidate genes (Nrf2, NGB, and SLC2A6) were correlated with SNAI3-AS1 (Fig. 4A and Supplementary Table 7). Following SNAI3-AS1 overexpression or knockdown, the mRNA levels of these three candidate genes were detected in glioma cells treated with DMSO and erastin. Results showed that there were no significant changes of NGB and SLC2A6 after SNAI3-AS1 regulation (Supplementary Fig. 6A, B). However, Nrf2 was downregulated after SNAI3-AS1 overexpression, and upregulated after SNAI3-AS1 knockdown under both DMSO and erastin treatments, which agreed with the trends in TCGA and CGGA693 databases (Fig. 4B, C). The following western blotting showed that overexpression and knockdown of SNAI3-AS1, respectively, resulted in an evident decrease and increase of Nrf2 protein levels after erastin treatment. The expression of ferroptosis indicators, GPX4 and 4-HNE, was altered correspondingly. Interestingly, no significant effects were observed in DMSO-treated cells (Fig. 4D). Nrf2 has been reported to be a key transcription factor of anti-ferroptosis. When iron-dependent lipid ROS accumulated, Nrf2 can detach from Keap1 to avoid ubiquitination-mediated degradation, and thus transcriptionally activate downstream effectors against ferroptosis, including SLC7A11, GPX4, and FTH1 et al. [14]. Accordingly, we supposed that SNAI3-AS1 was able to inhibit Nrf2 mRNA levels, but the regulation of SNAI3-AS1 in ferroptosis may depend on intracellular ferroptosis-inducing conditions due to the presence of ubiquitin–proteasome pathway mediated by Keap1. As expected, under erastin treatment, the downstream anti-ferroptosis effectors (SLC7A11, GPX4, and FTH1) regulated by Nrf2 were downregulated after SNAI3-AS1 overexpression, and upregulated after SNAI3-AS1 knockdown (Supplementary Fig. 6C). Moreover, we detected the correlations between SNAI3-AS1 and Keap1 in TCGA and CGGA693 databases (Supplementary Fig. 7A). We also found that SNAI3-AS1 overexpression or knockdown had no effect on the expression of Keap1 (Supplementary Fig. 7B, C). After treatment with CHX for the indicated time, SNAI3-AS1 did not change the half-life of Nrf2 protein (Supplementary Fig. 7D). These results fully illustrated that the regulation of SNAI3-AS1 on Nrf2 expression directly occurred at the mRNA level rather than at the protein level.

To further test how SNAI3-AS1 affected Nrf2 mRNA level, we first constructed a dual-luciferase reporter plasmid containing Nrf2 promoter. The assays showed no change in the transcriptional activity of Nrf2 promoter following SNAI3-AS1 overexpression and knockdown (Fig. 4E), which indicated that the mRNA alteration of Nrf2 may occur at the post-transcriptional level but rather at the transcriptional level. Therefore, we explored the effect of SNAI3-AS1 on the mRNA stability of Nrf2. Actinomycin D assays revealed that SNAI3-AS1 overexpression could markedly inhibit the mRNA stability of Nrf2, while SNAI3-AS1 knockdown resulted in increased mRNA stability of Nrf2 (Fig. 4F). Previous study has suggested that Nrf2 3’ UTR made a significant contribution to the mRNA stability [15]. Thus, we transfected a dual-luciferase reporter plasmid containing the Nrf2 mRNA 3’UTR into cells and found that the relative luciferase activity was repressed after SNAI3-AS1 overexpression, while increased after SNAI3-AS1 knockdown (Fig. 4G). Overall, SNAI3-AS1 regulates Nrf2 expression by inhibiting its mRNA stability in glioma cells.

### SNAI3-AS1 binds to SND1 protein in glioma cells

**Fig. 5:**
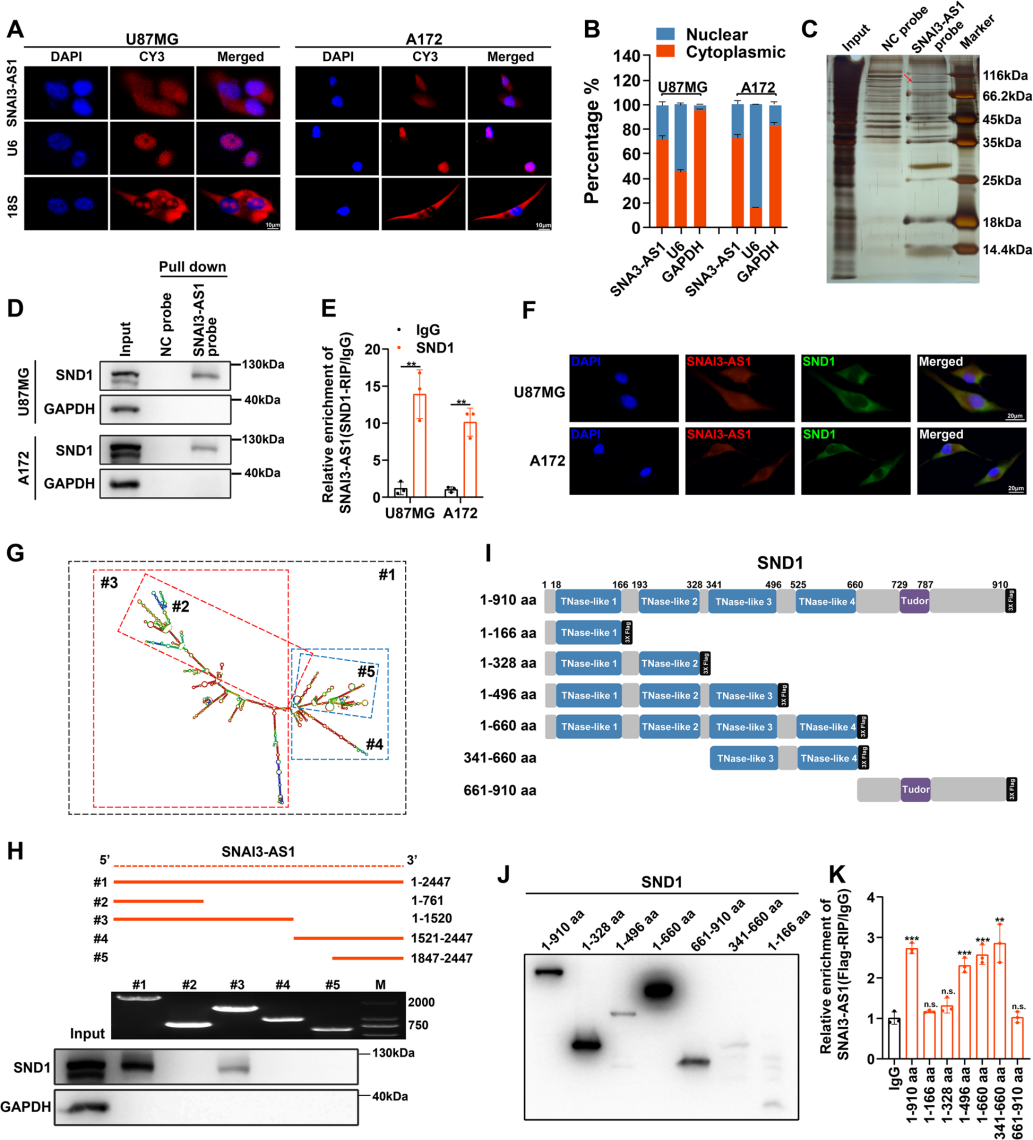
SNAI3-AS1 binds to SND1 protein.** A** RNA FISH analysis of SNAI3-AS1 localization in U87MG and A172 cells. 18S and U6 were used as positive controls. **B** After nucleocytoplasmic separation assay, the expression level of SNAI3-AS1 was determined via RT-qPCR. GAPDH and U6 were applied as positive controls. **C** Silver staining was used to identify the SNAI3-AS1-binding proteins pulled down by synthesized biotin-labeled SNAI3-AS1 probe. **D** Validation of the interaction between SNAI3-AS1 and SND1 protein through western blotting. **E** RIP assays were performed using anti-SND1 and IgG antibodies. The enrichments of SNAI3-AS1 by SND1 or IgG were detected via RT-qPCR. **F** The colocalization between SNAI3-AS1 and SND1 was determined by FISH combined with IF staining. **G** The predicted secondary structure of SNAI3-AS1. **H** Deletion mapping of the SND1-binding domain in SNAI3-AS1. Top, diagrams of full-length SNAI3-AS1 and the deletion fragments. Middle, the in vitro–transcribed full-length SNAI3-AS1 and deletion fragments with correct sizes were visualized by agarose gel image in U87MG cells. Bottom, immunoblot analysis for SND1 in the protein samples pulled down by different biotinylated SNAI3-AS1 truncations in U87MG cells. **I** The diagrams of Flag-tagged full-length or truncation plasmids with various assembled domains of SND1 protein. **J** Full-length or truncations of recombinant SND1 protein with correct sizes were validated by western blotting using anti-Flag in U87MG cells. **K** RT-qPCR detected the relative enrichment levels of SNAI3-AS1 in full-length or truncation SND1 RIP assays using anti-Flag and anti-IgG in U87MG cells. **P < 0.01, ***P < 0.001, and n.s., not significant

To explore the molecular mechanism of SNAI3-AS1 in regulating Nrf2 mRNA stability, we first determined the subcellular location of SNAI3-AS1. FISH and nucleocytoplasmic separation assays confirmed that SNAI3-AS1 was distributed in both the cytoplasm and nucleus, but is predominantly expressed in cytoplasm (Fig. 5A, B). It has been reported that lncRNA often regulates mRNA level via functioning as a miRNA sponge, and Ago2 protein is a mediator of lnRNA–miRNA interactions [16]. In order to verify whether SNAI3-AS1 regulates Nrf2 mRNA stability by sponging miRNAs, we conducted RIP experiments and found no detectable enrichment of SNAI3-AS1 in the Ago2 antibody group compared to the IgG control group (Supplementary Fig. 8A). In addition, lncRNA usually exerts its function through lncRNA-protein interactions. We next performed an RNA pulldown assay using in vitro synthesized biotin-labeled SNAI3-AS1 or NC probes. Silver staining and mass spectrometry was used to identify specific binding proteins (Fig. 5C and Supplementary Table 8). Among the top 10 possible candidate binding proteins, only SND1, a known RNA-binding protein, has been reported to be involved in the regulation of mRNA stability [17] (Supplementary Fig. 8B, C). The physical interaction between SNAI3-AS1 and SND1 was further confirmed by an RNA pull-down assay and RIP/RT-qPCR analysis (Fig. 5D, E). Moreover, a FISH/IF colocalization assay showed consistent result that SNAI3-AS1 and SND1 colocalized in the cytoplasm (Fig. 5F).

To further elucidate the structural determinants of interactions between SNAI3-AS1 and SND1, RNA pulldown assays were conducted by using truncated SNAI3-AS1 fragments and cell lysates. Deletion-mapping experiments combined with western blotting analysis revealed that the 762- to 1520-nt regions of SNAI3-AS1 were essential for binding SND1 (Fig. 5G, H). In addition, Flag-tagged full-length or truncation plasmids with various assembled domains of SND1 protein were constructed to perform protein domain mapping analysis (Fig. 5I, J). Results demonstrated that TNase-like 3 and TNase-like 4 domains of SND1 were indispensable for interaction with SNAI3-AS1 (Fig. 5K), which was consistent with the previous study [18]. Of note, SNAI3-AS1 and SND1 don’t affect the expression of each other (Supplementary Fig. 8D, E).

### SND1 recognizes Nrf2 mRNA and enhances its stability in an m^6^A-dependent manner

**Fig. 6:**
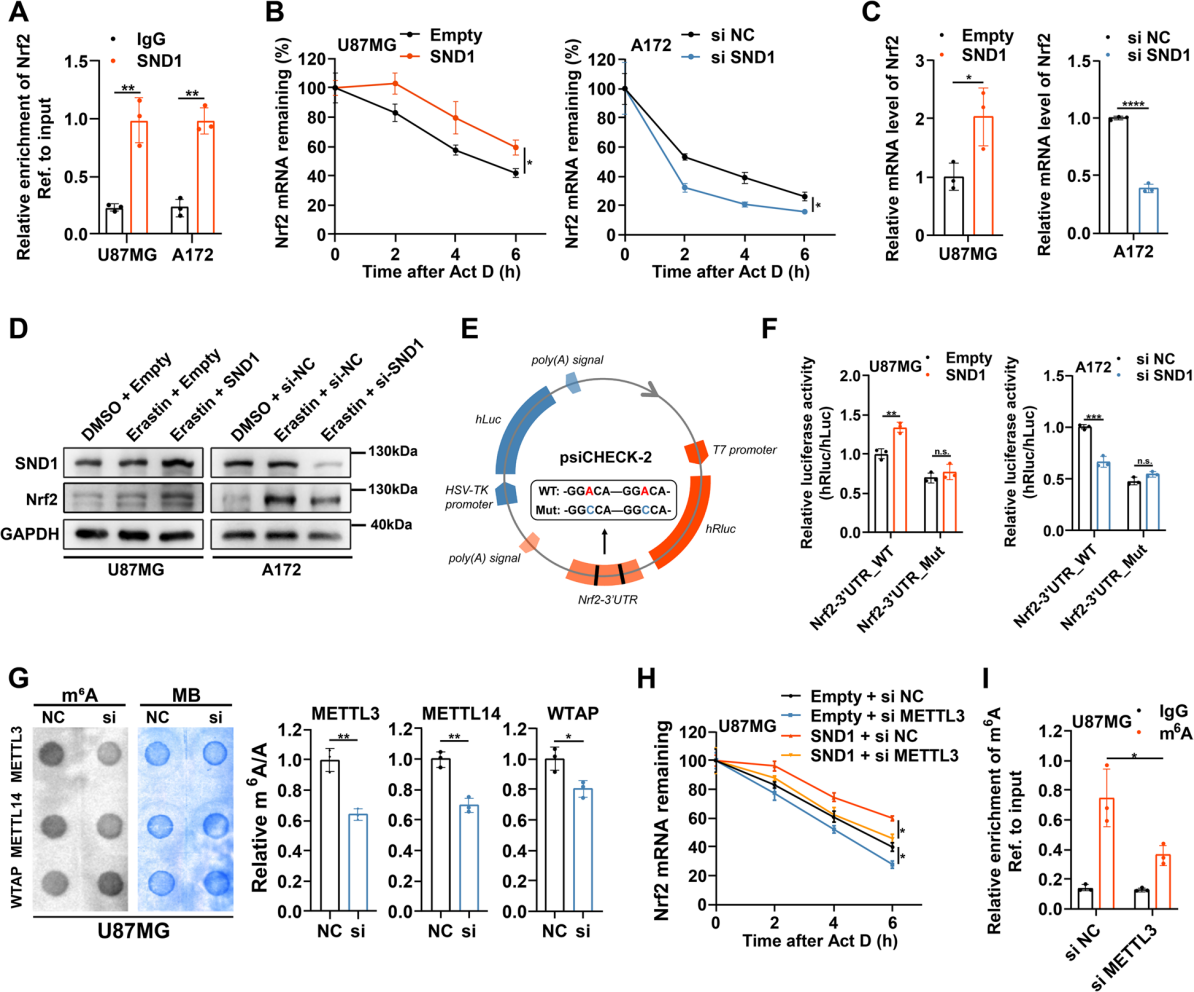
SND1 recognizes Nrf2 mRNA and enhances its stability in an m^6^A-dependent manner. **A** RIP assays were performed using anti-SND1 and IgG antibodies. The enrichments of Nrf2 mRNA by SND1 or IgG were detected via RT-qPCR. **B** After actinomycin D (5 μg/ml) treatment for 0, 2, 4, 6 h, RT-qPCR was used to analysis the Nrf2 mRNA stability in U87MG cells with SND1 overexpression and A172 cells with SND1 silence. **C** RT-qPCR detected the mRNA level of Nrf2 in U87MG cells with SND1 overexpression and A172 cells with SND1 silence. **D** Western blotting showed the protein levels of Nrf2 after SND1 overexpression or silence under (**E**) Schematic illustration was used to explain the design of dual luciferase reporter plasmids containing wild-type or mutant m^6^A sites in Nrf2 3’ UTR sequence. **F** Wild-type or mutant plasmids of reformed dual luciferase reporters were transfected into U87MG cells with SND1 overexpression and A172 cells with A172 cells with SND1 silence, respectively. The relative luciferase activity was measured and normalized. **G** m^6^A levels of U87MG cells with or without three m^6^A methyltransferases (METTL3, METTL14, and WTAP) silence were detected using the m^6^A RNA Methylation Quantification Kit and m^6^A dot blot assays. **H** U87MG cells with indicated interventions were treated with actinomycin D (5 μg/ml) for 0, 2, 4, 6 h, and Nrf2 mRNA stability was analyzed via RT-qPCR. **I** In METTL3-silenced or control U87MG cells, MeRIP assays and RT-qPCR were performed to calculate the relative enrichment of m^6^A modification. *P < 0.05, **P < 0.01, ***P < 0.001, ****P < 0.0001, and n.s., not significant

SND1, which is highly expressed in glioma (Supplementary Fig. 9A-C), has been reported to function as an m^6^A “reader” protein to bind and stabilize the m^6^A-modified RNAs [19]. There was a total of two m^6^A motifs (GGAC) at Nrf2 mRNA 3’UTR (Supplementary Fig. 9D). Previous studies suggested that the m^6^A modification occurring at Nrf2 mRNA 3’UTR was closely linked to the mRNA stability [20, 21]. We also observed a significant positive correlation between SND1 and Nrf2 in glioma according to the results of public databases (Supplementary Fig. 9E). Therefore, we speculated that SND1 might be involved in the regulation of Nrf2 mRNA stability. RIP/RT-qPCR analyses were then performed and demonstrated an obvious enrichment of Nrf2 mRNA in anti-SND1 group compared with anti-IgG group (Fig. 6A). This result indicated that SND1 was able to bind Nrf2 mRNA. Next, actinomycin D assays revealed that overexpressing SND1 could lead to a remarkable elevation of Nrf2 mRNA stability, and silencing SND1 exerted an opposite effect (Fig. 6B). We also observed consistent alterations of mRNA and protein levels of Nrf2 after SND1 overexpression or silence (Fig. 6C, D).

To confirm whether SND1 regulates Nrf2 mRNA in an m^6^A-dependent manner, we next constructed dual-luciferase reporter plasmids containing wild-type (Nrf2-3’UTR_WT) or mutant (Nrf2-3’UTR_MT) m^6^A sites in Nrf2 3’ UTR sequence (Fig. 6E). Luciferase reporter assays revealed that the relative luciferase activities of reporters with wild-type m^6^A sites, but not those with mutant m^6^A sites, were increased after overexpressing SND1, and were decreased after silencing SND1 (Fig. 6F). This indicated that SND1 regulated Nrf2 mRNA via the recognition of m^6^A sites in Nrf2 mRNA 3’ UTR sequence. It is well known that the m^6^A methylation level was directly determined by m^6^A methyltransferases, mainly including MELLT3, METTL14 and WTAP. After silencing METTL3, a more pronounced decrease of global m^6^A methylation level was observed compared with silencing METTL14 and WTAP in U87MG cells (Fig. 6G and Supplementary Fig. 9F). Moreover, public databases showed a significant positive correlation between METTL3 and Nrf2 (Supplementary Fig. 9G). Actinomycin D assays showed that silencing METTL3 blocked SND1 to accelerate Nrf2 mRNA stability (Fig. 6H). MeRIP-qPCR was applied to further confirm the m^6^A-dependent modification of Nrf2 mRNA. Results revealed that m^6^A methylation level of Nrf2 mRNA was obviously reduced around the putative m^6^A site after METTL3 silenced (Fig. 6I). All above results suggested that SND1 promoted Nrf2 mRNA stability in an m^6^A-dependent manner.

### SNAI3-AS1 perturbed the recognition of Nrf2 3’UTR by SND1 to exert ferroptosis-sensitizing activity

**Fig. 7:**
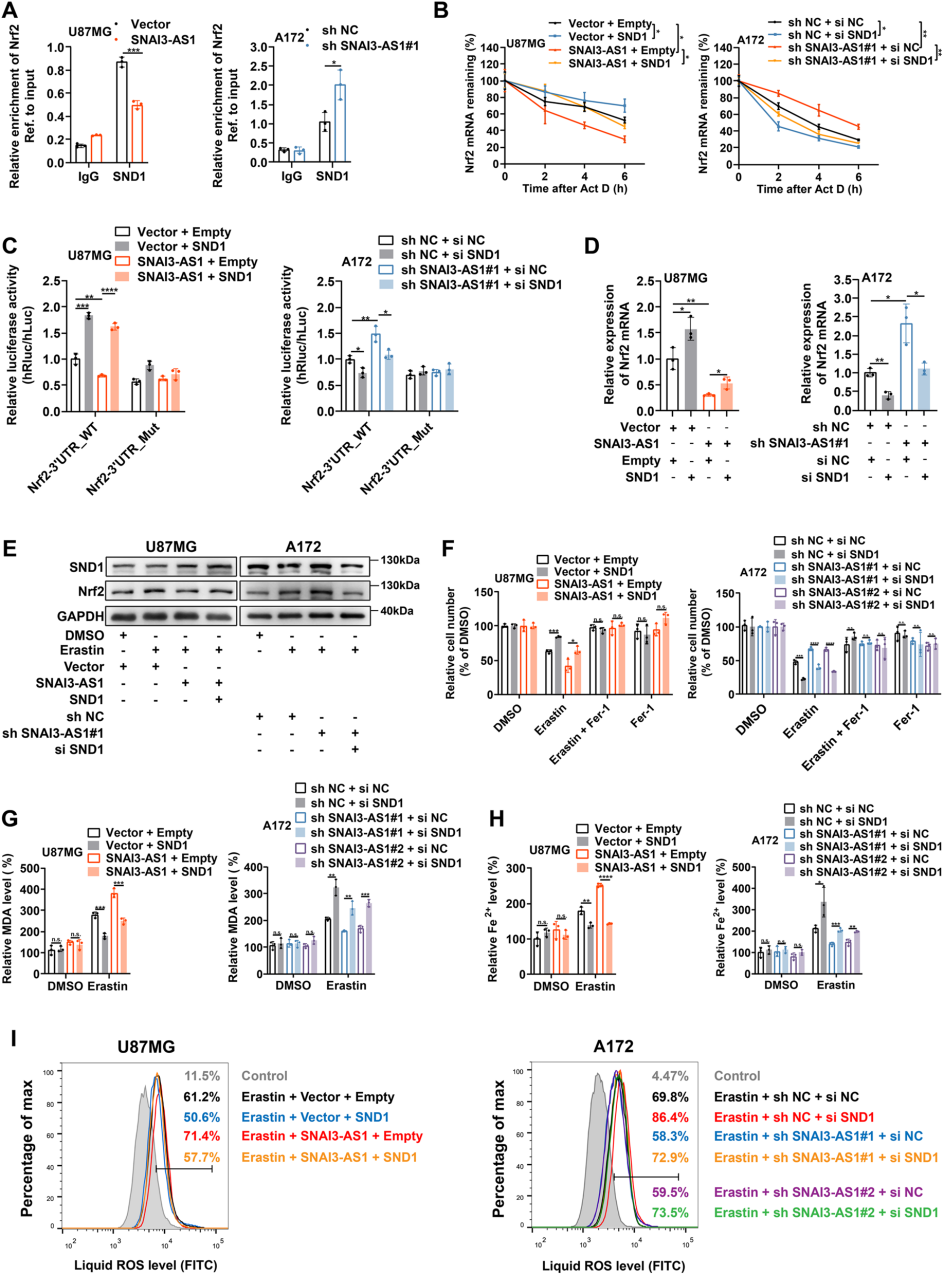
SNAI3-AS1 perturbed the recognition of Nrf2 3’UTR by SND1 to exert ferroptosis-sensitizing activity.** A** RIP assays showed the enrichments of Nrf2 mRNA by SND1 in Nrf2 mRNA in U87MG cells with stable SNAI3-AS1 overexpression or A172 cells with stable SNAI3-AS1 knockdown. **B** After actinomycin D (5 μg/ml) treatment for 0, 2, 4, 6 h, RT-qPCR was used to analysis the Nrf2 mRNA stability in U87MG and A172 cells with indicated interventions. **C** Dual luciferase reporter plasmids containing Wild-type or mutant Nrf2 mRNA 3’UTR p were transfected into U87MG and A172 cells with indicated interventions, respectively. The relative luciferase activity was measured and normalized. **D** RT-qPCR detected the mRNA level of Nrf2 in U87MG and A172 cells with indicated interventions. **E** After DMSO or erastin (10 μM, 48 h) treatments, the protein levels of Nrf2 in U87MG and A172 cells with indicated interventions were determined by western blotting. **F-I** U87MG and A172 cells with indicated interventions were treated with erastin (10 μM) ± ferrostatin-1 (2 μM) for 48 h, cell viabilities were detected via CCK8 assays **(F)**, intracellular MDA was determined by MDA assays (**G**), intracellular Fe^2+^ was measured by iron detection assays (**H**), lipid ROS accumulation was analyzed by flow cytometry with C11-BODIPY staining (**I**). *P < 0.05, **P < 0.01, ***P < 0.001, ****P < 0.0001, and n.s., not significant

Given that SNAI3-AS1 could inhibit Nrf2 mRNA stability, while SNAI3-AS1-interacted SND1 promoted Nrf2 mRNA stability via m^6^A-dependent recognition, we speculated that SNAI3-AS1 might bind SND1 to perturb its recognition of Nrf2 mRNA. RIP/RT-qPCR analysis revealed that Nrf2 mRNA binding in SND1 in U87MG cells were appreciably decreased by SNAI3-AS1 overexpression, and increased by SNAI3-AS1 knockdown (Fig. 7A). Actinomycin D assays showed that the inhibitory effect of SNAI3-AS1 overexpression on the stability of Nrf2 mRNA was greatly restored after SND1 overexpression, and silencing SND1 also impaired the promotion of Nrf2 mRNA stability by SNAI3-AS1 knockdown (Fig. 7B). Furthermore, we performed dual-luciferase reporter assays and found that SND1 overexpression could rescue SNAI3-AS1-mediated decrease of relative luciferase activity of Nrf2 mRNA 3’UTR, and SND1 silence impaired the increased luciferase activity of Nrf2 mRNA 3’UTR caused by SNAI3-AS1 knockdown (Fig. 7C). Both RT-qPCR and western blotting exhibited the consistent restorations on Nrf2 expression by SND1 overexpression or silence (Fig. 7D, E). These data suggested that SNAI3-AS1 competitively binds to SND1 and perturbed its recognition of Nrf2 3’UTR, thereby decrease the stability of Nrf2 mRNA.

Next, we designed rescue experiments to validate whether SND1 is involved in the ferroptosis-sensitizing activity of SNAI3-AS1. We found that SND1 overexpression significantly suppressed the erastin-induced ferroptotic cell death and impaired the promoting effect of SNAI3-AS1 overexpression, while SND1 silence significantly promoted the erastin-induced ferroptotic cell death and restored the inhibitory effect of SNAI3-AS1 knockdown (Fig. 7F). Moreover, SND1 overexpression limited the facilitation effects of SNAI3-AS1 overexpression on the accumulation of MDA, Fe^2+^, and lipid ROS, and SND1 silence partially restored the decreased levels of MDA, Fe^2+^, and lipid ROS caused by SNAI3-AS1 knockdown (Fig. 7G-I). Taken together, SND1 is the key intermediate protein for SNAI3-AS1-regulating increased sensitivity to ferroptotic challenge.

### SNAI3-AS1 enhances the anti-tumor activity of erastin by promoting ferroptosis in vivo

**Fig. 8:**
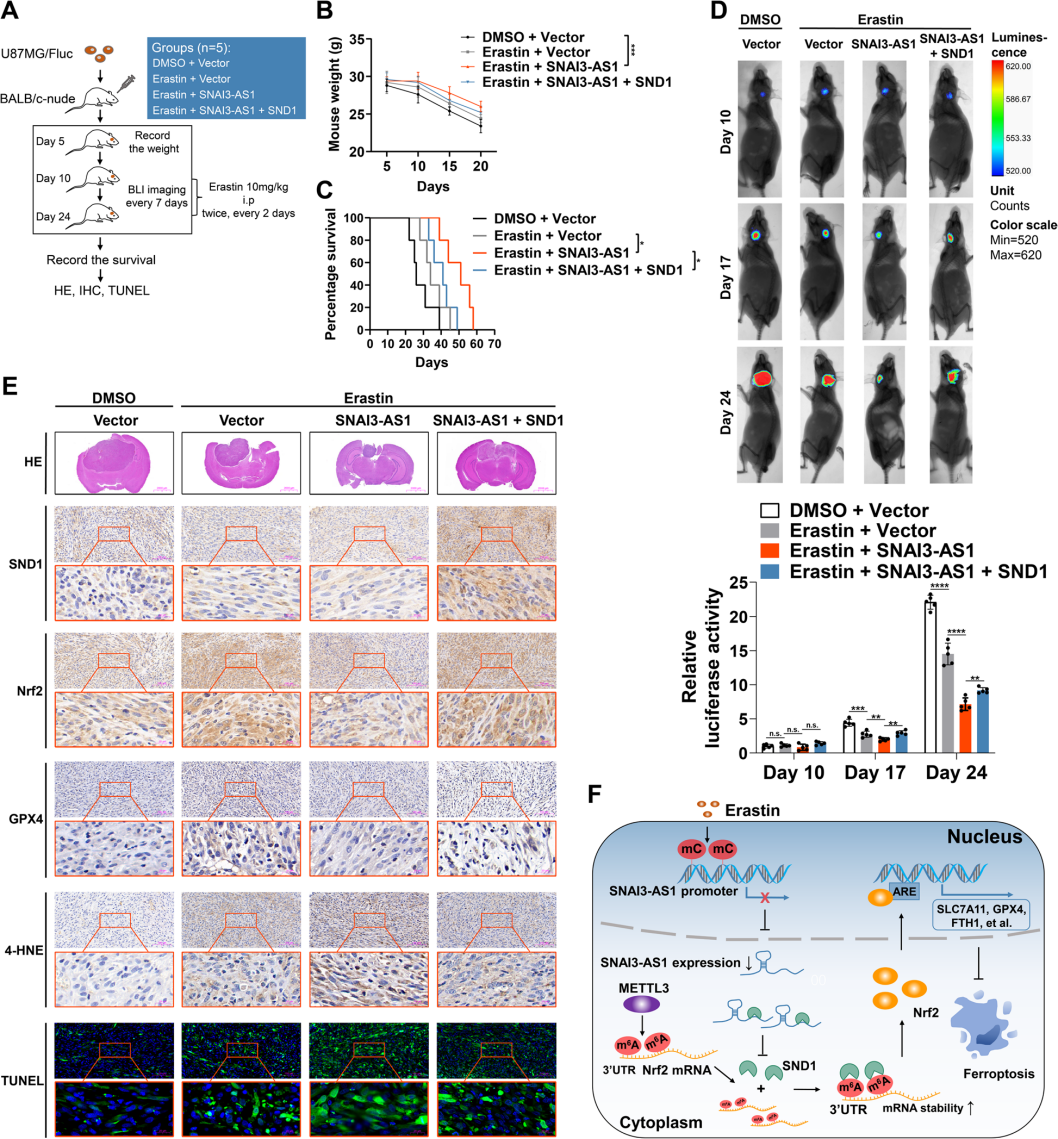
SNAI3-AS1 overexpression promotes erastin-induced ferroptosis in vivo. **A** Schematic illustration showing the design of animal experiments. **B** Body weights of mice in each group were recorded during the experiment. **C** Kaplan–Meier survival of mice in each group. **D** Representative bioluminescence images and performed on days 10, 17, and 24 after implantation. Down, statistical analysis of bioluminescent tracking plots. **E** Representative images of HE staining, IHC assays (SND1, Nrf2, GPX4 and 4-HNE), and TUNEL assays in each group. **F** Schematic diagram of SNAI3-AS1/SND1/Nrf2 axis regulating ferroptosis in glioma cells. Erastin inhibits SNAI3-AS1 by increasing DNA methylation level of SNAI3-AS1 promoter. Decreased expression of SNI3-AS1 favors SND1 to bind and stabilize Nrf2 mRNA in an m.^6^A-dependent manner, thereby suppresses ferroptotic cell death. *P < 0.05, and ***P < 0.001

To determine whether the ferroptosis-sensitizing activity of SNAI3-AS1 remained in vivo, we constructed mouse orthotopic models via U87MG/Fluc cells. Ten days after implantation, BLI imaging was performed to confirm tumorigenesis. Then, mice were injected intraperitoneally with erastin (10 mg/kg per mouse) or DMSO (0.3%) twice, every 2 days (Fig. 8A). With tumor progression, mice manifested loss of body weight, which was appreciably relieved by combined erastin treatment and SNAI3-AS1 overexpression (Fig. 8B). Kaplan–Meier analysis of mice survival indicated that erastin treatment with SNAI3-AS1 overexpression dramatically prolonged the survival time, which was attenuated by SND1 overexpression (Fig. 8C). BLI imaging exhibited that tumor inhibition was more prominent when erastin treatment combined with SNAI3-AS1 overexpression, and such effect was impaired followed by SND1 overexpression (Fig. 8D).

Next, we sought to determine whether the regulatory relationship among SNAI3-AS1/SND1/Nrf2 pathway existed in vivo. Consistent with observed in vitro, when erastin was paired with SNAI3-AS1 overexpression, the expressions of Nrf2 and GPX4 was significantly reduced, which was restored by SND1 overexpression. According to IHC assays for 4-HNE and TUNEL assays, mice with SNAI3-AS1 overexpression displayed increased lipid peroxidation and more cell death, while SND1 overexpression limited these effects (Fig. 8E). Overall, SNAI3-AS1 enhances the anti-tumor activity of erastin by promoting ferroptosis in vitro.

## Discussion

LncRNAs have been widely reported for its regulatory role in various biological processes of cancer. LncRNAs dysregulation is generally involved in pertinent tumor cellular events, including resistance to cell death [22]. Ferroptosis, an emerging field in cell death, has garnered enormous attention from researchers. Distinctive from other forms of cell death, ferroptosis is characterized by the accumulation of iron-dependent lipid peroxidation causing cell damage and death. Ferroptosis follows multiple subroutines, including iron metabolism, glutathione metabolism, and lipid metabolism, each of which has specific molecular cascades and regulatory pathways [23]. Although the critical signaling pathways of ferroptosis has become gradually clear, the role and mechanism of lncRNAs in ferroptosis remain unclear and deserves deep explorations.

Only an extremely low amounts of studies have revealed the relationship and mechanism between lncRNAs and ferroptosis in glioma [24, 25]. In this study, we demonstrated that lncRNA SNAI3-AS1 was dramatically downregulated during erastin-induced ferroptosis by elevated DNA methylation level at the CpG islands within its promoter. SNAI3-AS1 could increase the sensitivity of glioma cells to ferroptosis. Mechanistically, SNAI3-AS1 reduced Nrf2 mRNA stability through competitively binding with SND1, which was a m^6^A reader with Nrf2 mRNA-stabilizing activity by recognizing the METTL3-mediated m^6^A modification (Fig. 8F).

Previous studies have revealed that SNAI3-AS1 is upregulated in HCC, and can promote tumor cell proliferation and metastasis by activating UPF1/Smad7 signaling pathway and by acting as a sponge for miR-27-3p and miR-34a-5p to upregulate PEG10 [26, 27]. Here, we interestingly found that SNAI3-AS1 functions as a tumor suppressor in glioma. More importantly, SNAI3-AS1 is tightly correlated with ferroptotic cell death. We first observed that elevated DNA methylation level led to the downregulation of SNAI3-AS1 expression in erastin-induced ferroptosis. Epigenetic alterations may represent a way by which tumor cells cope with various cellular stresses [28]. Zhang et al.’s study also found that enhanced transcriptional activity of p53 upregulates the expression of lncRNA NEAT1 in erastin-induced ferroptosis [29]. These motivated us to further explore the certain role and mechanism of SNAI3-AS1 in ferroptosis. Next, we confirmed that SNAI3-AS1 can increase the sensitivity of glioma cells to ferroptosis by reducing Nrf2 mRNA stability. Nrf2, encoded by the NFE2L2 gene, is a key transcription factor that governs cellular responses to oxidative stress by transcriptional activation of multiple downstream genes containing antioxidant response element (ARE) consensus sequences [30, 31]. Importantly, Nrf2 has also been verified to be a negative regulator of ferroptosis via the induction of multiple components of the ferroptosis cascade, including SLC7A11, GPX4, and FTH1 et al. [14, 32]. Sensitizing tumor cells to ferroptotic cell death by targeting Nrf2/ARE signaling pathway has been regarded as a promising synergistic strategy with chemoradiotherapy or immunotherapy [33–35]. Our in vitro and in vivo results indicated that inhibiting Nrf2/ARE signaling pathway by overexpressing SNAI3-AS1 dramatically enhanced erastin-induced ferroptosis in glioma and suppressed tumor growth accordingly. Our findings may provide a novel insight into reinforcing anti-tumor responses by effective ferroptosis induction.

To date, although the mechanisms of lncRNAs involvement in Nrf2 regulation have been extensively investigated, previous studies focus primarily on the post-translational regulation of Nrf2 through Keap1-mediated ubiquitination pathway [36, 37]. For instance, Bi et al. demonstrated that miR-6077 induced upregulation of NRF2 by targeting Keap1 [38]. Han et al. reported that lncRNA LINC00239 inhibits ferroptosis in colorectal cancer by binding to Keap1 to stabilize Nrf2 protein [39]. Compared with Nrf2 regulation at post-translational level, regulations of Nrf2 occurred at mRNA levels require to be explored more extensively. It is well known that lncRNAs usually serve as miRNA sponges to regulate downstream mRNAs. Besides that, binding to RNA binding protein (RBP) and affect its activity is another common mechanism that lncRNAs participate in the regulation of downstream mRNAs [40]. For example, Fang et al.’s work demonstrated that the lncRNA LINC00525 reduced p21 mRNA stability through competitive binding with RBMS2 [41]. Here, we uncovered a novel post-transcriptional mechanism that SNAI3-AS1 competitively binds to SND1, thus reduces the stability of Nrf2 mRNA, which enriched the understanding of the regulatory mechanisms of Nrf2.

Alternately, it is worth noting that m^6^A modification plays a critical role in the regulatory relationship between SND1 and Nrf2. RNA m^6^A modification referring to the methylation at the N6 position of adenosine, has been well documented to be able to control many facets of RNA biology [42]. Particularly, m^6^A modifications enriched in 3’-UTR generally determine RNA stability under the recognition of m^6^A “reader” proteins [43]. Our results suggested that SND1, which has been reported as a m^6^A “reader” protein [19], can recognize the m^6^A modifications in Nrf2 3’-UTR and thus promotes mRNA stability of Nrf2. In addition, we found that the m^6^A methyltransferase METTL3 mediates m^6^A modifications in Nrf2 3’-UTR. Ye et al. also confirmed that Nrf2 mRNA level was regulated by m^6^A modifications in hypopharyngeal squamous cell carcinoma (HPSCC) [21]. Interestingly, their findings revealed that m^6^A demethylase ALKBH5 promotes HPSCC cells to ferroptosis by reducing m^6^A modifications in Nrf2 3’-UTR and thus dysregulates Nrf2 expression in an m^6^A-IGF2BP2-dependent mechanism. In accordance with this, whether such regulation of Nrf2 by ALKBH5 and IGF2BP2 also has an influence on ferroptotic cell death in glioma needs to be further investigated.

## Conclusions

In summary, we illustrated that lncRNA SNAI3-AS1 perturbs the m^6^A-deependent recognition of Nrf2 3’UTR by SND1 to reduce mRNA stability of Nrf2, thereby enhancing the anti-tumor activity of erastin by promoting ferroptosis in glioma. These findings indicate that SNAI3-AS1 may serve as a target for ferroptosis-dependent therapy in glioma.

## Supplementary Information

**Additional file 1:** **Supplementary Table 1.**Clinical information of patients with glioma. **Supplementary Table 2.** Plasmid vector sequence and siRNA used in this study. **Supplementary Table 3**. Primers for RT-qPCR. **Supplementary Table 4.** **Supplementary Table 5.** **Supplementary Table 6.** 

**Additional file 2: Supplementary Table 7.** Correlation between SNAI3-AS1 and ferropotsis related genes in TCGA and CGGA693 databases.

**Additional file 3:** **Supplementary Table 8.** Mass spectrometry to identify SNAI3-AS1 binding proteins.

**Additional file 4:** **Supplementary Fig. 1.** The expression of eight candidate lncRNAs in U87MG, U251 and A172 cells under erastin (5 μM, 48 h) treatments as measured by RT qPCR. *P < 0.05, and n.s., not significant.

**Additional file 5:** **Supplementary Fig. 2.** (A-R) Correlation between SNAI3-AS1 expression and 18 CpG sites of SNAI3-AS1 DNA promoter. (S) CG islands distribution of BSP.

**Additional file 6:** **Supplementary Fig. 3.** (A-B) Comparing the expression of SNAI3-AS1 between normal tissues and different WHO grade tissues based on TCGA and CGGA693databases. (C) SNAI3-AS1 expression was determined in 24 paired glioma tissues and nontumoral brain tissues via RT-qPCR. NBT, nontumoral brain tissue. (D) SNAI3-AS1 expression was determined in normal astrocyte and several glioma cell lines via RT-qPCR. (E-F) Kaplan–Meier analysis based on SNAI3-AS1 expression in glioma using the data from TCGA and CGGA693 databases. (G-H) The receiver operating characteristic (ROC) curves of SNAI3-AS1 in glioma using the data from TCGA and CGGA693 databases. *P < 0.05, ***P < 0.001, ****P < 0.0001, and n.s., not significant.

**Additional file 7:** **Supplementary Fig. 4.** (A) RT-qPCR was used to detect the expression of SNAI3-AS1 in A172 cells transfected with two sh SNAI3-AS1 lentiviral vectors or control vector. (B) The growth curves of transfected A172 cells were determined by CCK8 assays. (C) The colony formation assays were performed in transfected A172 cells. (D) The proliferation of transfected A172 cells was detected by EdU staining assays. (E) The transwell assays showed the migration and invasion abilities of transfected A172 cells. (F) Cell cycle distributions of transfected A172 cells were measured by flow cytometry. *P < 0.05, **P < 0.01, ***P < 0.001, and ****P < 0.0001.

**Additional file 8:** **Supplementary Fig. 5.** (A-F) U87MG and U251 cells stably overexpressing SNAI3-AS1 were treated with erastin (10 μM) ± Deferoxamine (DFO, 100 μM) for 48 h, different concentration of erastin (5/10/20 µM) for 48 h, or 10 µM erastin for 24/48/72 h. Cell viabilities were detected via CCK8 assays. (G-I) A172 cells with stable SNAI3-AS1 knockdown were treated with erastin (10 μM) ± Deferoxamine (DFO, 100 μM) for 48 h, different concentration of erastin (5/10/20 µM) for 48 h, or 10 µM erastin for 24/48/72 h. Cell viabilities were detected via CCK8 assays. *P < 0.05, **P < 0.01, ***P < 0.001, ****P < 0.0001, and n.s., not significant.

**Additional file 9:** **Supplementary Fig. 6.** RT-qPCR was used to detect the mRNA levels of NGB (A) and SLC2A6 (B) after SNAI3-AS1 overexpression or knockdown under DMSO and erastin (10 μM, 48 h) treatments. (C) The mRNA levels of SLC7A11, GPX4, and FTH1 were measured in glioma cells with SNAI3-AS1 overexpression or knockdown and erastin (10 μM, 48 h) treatments. **P < 0.01, ***P < 0.001, ****P < 0.0001, and n.s., not significant.

**Additional file 10:** **Supplementary Fig. 7.** (A) The correlations between SNAI3-AS1 and Keap1 in TCGA and CGGA693 databases. (B) The mRNA level of Keap1 detected by RT-qPCR after SNAI3-AS1 overexpression or knockdown. (C) The protein level of Keap1 detected by WB after SNAI3-AS1 overexpression or knockdown. (D) The Nrf2 protein level in indicated time point after treated with cycloheximide (CHX, 10 µg/ mL) in glioma cells with SNAI3-AS1 overexpression or knockdown. n.s., not significant.

**Additional file 11:** **Supplementary Fig. 8.** (A) RIP assays were performed using anti-Ago2 and IgG antibodies. The enrichments of SNAI3-AS1 by Ago2 or IgG were detected via RT-qPCR. (B) Top 10 possible candidate binding proteins of SNAI3-AS1 based on the mass spectrometry analysis. (C) Mass spectrometry revealed SND1 peptides pulled down by SNAI3-AS1 probe. (D) The expression of SNAI3-AS1 detected by RT-qPCR after overexpressing SND1 or silencing SND1. (E) Western blotting showed the expression of SND1 protein after SNAI3-AS1 overexpression or knockdown. n.s., not significant.

**Additional file 12:** **Supplementary Fig. 9.** (A) Comparing the expression of SND1 between normal tissues from GTEx database and glioma tissues from TCGA database. (B) SND1 expressions in a paired glioma tissue and nontumoral brain tissue showed by IHC. (C) SND1 expressions in 4 paired glioma tissue and nontumoral brain tissue showed by western blotting. (D) The sequences of Nrf2 mRNA 3’UTR. (E) The correlation between SND1 and Nrf2 in TCGA and CGGA693 databases. (F) Silencing METTL3, METTL14, and WTAP in U87MG cells were verified by western blotting. (G) The correlation between METTL3 and Nrf2 in TCGA and CGGA693 databases.

## Data Availability

The datasets used and/or analyzed during the current study are available from the corresponding author on reasonable request.

## Abbreviations

lncRNA: Long non-coding RNA
ATCC: American Type Culture Collection
FBS: Fetal bovine serum
BSP: Bisulfite sequencing PCR
RIP: RNA immunoprecipitation
FISH: Fluorescent in situ hybridization
IHC: Immunohistochemistry
TUNEL: Terminal deoxynucleotidyl transferase dUTP nick end labeling
MeRIP: Methylated RNA immunoprecipitation
MDA: Malondialdehyde
Fluc: Firefly luciferase
TCGA: The Cancer Genome database
CGGA693: Chinese Glioma Genome Atlas mRNAseq_693 database
GTEx: Genotype-Tissue Expression
ROC: Receiver operating characteristics
ARE: Antioxidant response element
RBP: RNA binding protein
