# Supplementary material for: Epigenetically silenced lncRNA SNAI3-AS1 promotes ferroptosis in glioma via perturbing the m6A-dependent recognition of Nrf2 mRNA mediated by SND1
Source: J Exp Clin Cancer Res. 2023 May 19;42:127. doi: 10.1186/s13046-023-02684-3 (PMC10197824; doi:10.1186/s13046-023-02684-3)
Supplement: Supplementary file 1 — Additional file 1: Supplementary Table 1.Clinical information of patients with glioma. Supplementary Table 2. Plasmid vector sequence and siRNA used in this study. Supplementary Table 3. Primers for RT-qPCR. Supplementary Table 4. Supplementary Table 5. Supplementary Table 6. [file 13046_2023_2684_MOESM1_ESM.docx]

**Supplementary Table 1. Clinical information of patients with glioma**

| **No.** | **Age** | **Gender** | **Pathological diagnosis** | **WHO grade** |
| --- | --- | --- | --- | --- |
| 1 | 51 | Male | Oligoastrocytoma | 2 |
| 2 | 47 | Female | Glioblastoma | 4 |
| 3 | 39 | Female | Glioblastoma | 4 |
| 4 | 29 | Male | Oligoastrocytoma | 3 |
| 5 | 64 | Female | Astrocytoma | 2 |
| 6 | 61 | Male | Oligoastrocytoma | 3 |
| 7 | 57 | Male | Astrocytoma | 3 |
| 8 | 51 | Male | Oligoastrocytoma | 2 |
| 9 | 56 | Male | Astrocytoma | 2 |
| 10 | 72 | Female | Glioblastoma | 4 |
| 11 | 61 | Male | Astrocytoma | 3 |
| 12 | 62 | Female | Glioblastoma | 4 |
| 13 | 45 | Female | Astrocytoma | 4 |
| 14 | 48 | Female | Oligoastrocytoma | 2 |
| 15 | 49 | Male | Astrocytoma | 4 |
| 16 | 48 | Male | Astrocytoma | 2 |
| 17 | 57 | Female | Astrocytoma | 2 |
| 18 | 56 | Male | Astrocytoma | 2 |
| 19 | 62 | Male | Oligoastrocytoma | 2 |
| 20 | 44 | Male | Glioblastoma | 4 |
| 21 | 54 | Female | Astrocytoma | 4 |
| 22 | 61 | Male | Glioblastoma | 4 |
| 23 | 60 | Female | Glioblastoma | 4 |
| 24 | 71 | Male | Oligoastrocytoma | 3 |

**Supplementary Table 2. Plasmid vector sequence and siRNA used in this study**

| **Name** | **Sequence (5’ - 3’)** | **Company** |
| --- | --- | --- |
| lenti-CMV-H_SNAI3-AS1-PGK-Puro | NR_024402.2 (2447 bp) | Genomeditech |
| lenti-CMV-H_SND1-PGK-Puro | NM_014390.4 (3448 bp) | Genomeditech |
| sh NC (PGMLV-SC5) | TTCTCCGAACGTGTCACGT | Genomeditech |
| H_SNAI3-AS1-shRNA1(PGMLV-SC5) | GCCTCTGCGTTATGTCGTTTG | Genomeditech |
| H_SNAI3-AS1-shRNA2(PGMLV-SC5) | GCCATCCCGAAACTGTGATTG | Genomeditech |
| si SND1 | GAAGGCAUGAGAGCUAAUAAU | QijingBio |
| si METTL3 | GCCUUAACAUUGCCCACUGAU | GeneCreate |
| si METTL14 | GCUAAUGUUGACAUUGACUUA | GeneCreate |
| si WTAP | GUUAUGGCAAGAGAUGAGUUA | GeneCreate |

**Supplementary Table 3. Primers for RT-qPCR**

| **Name** | | **Forward primer (5’ - 3’)** | **Reverse primer (5’ - 3’)** |
| --- | --- | --- | --- |
| H_SNAI3-AS1 | | GCGTTATGTCGTTTGGTTGATG | TGGCAGGAATGAGGTGAGC |
| H_GDNF-AS1 | | CGGAGTTCTAGCACCGAGA | AGTTAACCTCCCTGTGGCCT |
| H_WDFY3-AS2 | | TTGGGGCACTCATCCCATTC | TTGAGCTCGGACTGTGCATT |
| H_CPB2-AS1 | | GCCTAGTAGGGGGACTTCCA | TCCATCCTTCCCCGCTAAGA |
| H_SBF2-AS1 | | CACGACCCAGAAGGAGTCTAC | CCCGGTACCTTCCTGTCATA |
| H_PAXIP1-AS2 | | TGACCGAACGCTGAATGTGA | CTCCTGTGAACCACAGCACA |
| H_SNHG18 | | TGTGGCAGCCCACTCTATTG | TGGTGGACTTGAGTGGAAGC |
| H_PVT1 | | GGGGAATAACGCTGGTGGAA | CCCATGGACATCCAAGCTGT |
| H_Nrf2 | CGACGGAAAGAGTATGAG | | CTAGTTGTAACTGAGCGAAA |
| H_Keap1 | CAACTTCGCTGAGCAGATTGGC | | TGATGAGGGTCACCAGTTGGCA |
| H_NGB | AGTCCCTTCACTGCTTTCCTG | | GAAACCGACTCTGTCAGGGC |
| H_SLC2A6 | CCTGCAGTCCATCTTCGACA | | AAACATGATGGCCGCTGAGA |
| H_SLC7A11 | GTCTGGGTGGAACTCCTCAT | | CTCCAGCTGACACTCATGCTA |
| H_GPX4 | TGCTCTGTGGGGCTCTGG | | TCCTTGGCGGAAAACTCG |
| H_FTH1 | GCCAGAACTACCACCAGG | | TTCAAAGCCACATCATCG |
| H_GAPDH | ATCCCATCACCATCTTCC | | ATGACCCTTTTGGCTCCC |
| H_β-actin | CATGTACGTTGCTATCCAGGC | | CTCCTTAATGTCACGCACGAT |
| H_U6 | CTCGCTTCGGCAGCACA | | AACGCTTCACGAATTTGCGT |
| H_SNAI3-AS1-BSP | GAGGGAGGGATATAGGGTTTCGG | | TACGCGTTTATAACTCCAACTACTCG |
| H-Nrf2-MeRIP | TTCTTAGGACACCATTTGGGCTAGTT | | TGTCCATACAGTATTTATAAAAAAGTACATAGTGGTTAG |

**Supplementary Table 4**

| **SNAI3-AS1 promoter sequence (Dual-luciferase reporter)** |
| --- |
| ATATCTAGAATACATATTTTATTTTTTTTCAGTAGAGACGGGGGTTTCACCATGTTGGCCAAGCTGGTCTCAAACTCCTGACCTCAGGTGATCCGCCCACCTCAGCCTCCCCAAGTGCTGGGATTACAGGCTTGAGCCAGCGTGCCCAGCCTAGAATGTGTAATGAACTCTCCAAACTTATAGTAAAAAAGCAAACCATCCAGTTCGAGCGTGGACACAGAATATGAAGAGAGATTTCACCAAGAAAACCCACAGATGGCAAATAAGCACAAAGAAAGATGCTCCACGTCACTGGCCAACAGGGAAACGCGAATGAGGACCACAGCACCCTGCCAGCACATCCCTACCAGGACGCGGCTAAAATGAAAAACAGCAACTCCACCAAATGCTGGTGAGGATGCGGAAAATCTGCATCACCGTAGGGATGCAAAATGGCCCAGCCACTCTGAAAAACAATTAGACAGTTTCTTTAAAAAAAAAAAGTATATATATAATATATATATGTATATATACACACACATACATCTATATAGGTGTATACATATACACACACATATATCTATACAGGTGTATATATATATACACACACACAAACATACATATATACATAGAAAGAGAGAGAGAGACAGAGAGGCATATATATATATCTACCATCTGACCCAGCAACCGCACTCCTGGGCATTTATCCCAGAAGAATGGAAATGTATGTTCACACAAAAACCTGTATACAACATTTTATGGCAGCTTTATTGGTAAGGGAAACACAAAAAAATTAGCCAGTCGTGGTGGCGGCTTCTGGAAAGAAAAGAAAGAAAGAAAGAAAACGTTAGATGCTCTTTGCGGGGTGAGGCCTTGGCTAGAGAGAGGGTGGTCCATGGATTCCACGGACTGGCTCAGCAACAGAGGGGCAAAGCACTGATACAATCCCGGGGCACGTCCACATTCTATGCTGAATGAAATAAGCCAAACTCAAAAGATACTGGGTGGGCCACATAACTGCGATTCCAGTTTCAGAACATTTTGAAATGACGTTACAGAAATGAGTGGTCAGTGGCGGCCAGGTAACCCCGGCGCGAGGGGAGGGGCGGGGCTGTGAACGCACAGGTGCGGACCGGCCTCCCCGGCACGGATGTGGGCCTGCGGGCCCCCCATTACCGCTCCTTGGAGGTTACCGCTGGGAGAAACGGGGCGAGGGAGGGACACAGGGTCTCGGGCTGGGGGAGCAGTGGCGCGATTTCCGCTCACTGCAGCCTCGACCTCCCGGGCTCTGGCCATCCTCCCGCCTCAGCCTCCCGAGTAGCTGGAGCCACAAACGCGCACCACCACGGCGGATTCTTACGATTCATGGGAATCGATGATTGATGATTTCGATAGTTCGGTAATCGCTAATGGGCAGGCGCCGGGCGACCCTGCAGGGGCGGCAGCCGTCGCGGGGGAACGGGTGGCGCCGAGCTTGTCACGCGAAGGAGCGCGCGGCCCCGCCCGACCACCGCCGCGCCCCTCCCTGAGCCTCAGTTTCCCCGTCTGTCGAGGCCCTGGGAGGGCAGGACGGAGCGCGCCGCCGAATCAGCGCGCGACCCCCGCGTACCCGGCCTCGCTCCCGGCCATCCCCGTCCCAGGCCGGCCCGCGGCACTCACAGTACTTGATGACCGCGATGTTGACCGGCGCTGTACAAGTGACTGCCGCCAGCGGCTTCTCCGAGGCCATGGTCCCACCGCGCAGTGACCCCAGCTCCACAGCCACTTACGGCCCGCGATCCCACCCCAGAGGCCTCTGCCTCCTCACGCGCTACCACAGGATTGGCCCGTGCGAGCCCGGCTGCTCGCGCGCCGACCAATCAGCGGCGAGTCGGGCGCACGCATTACCTCTCAGCCAATGGACGGGATCGGACGATGGGTTGCTGACGTAGGTCCGCCCCACCCGCAGGGTGCGCTCGCCAGGCCTGGGGCGTGGTGTTGTCTGCGCAGGCGCCGTGTGCCCAGGCGCCTGGCGT |

**Supplementary Table 5**

| **Nrf2 promoter sequence (Dual-luciferase reporter)** |
| --- |
| GGGGTTCCCTTTATTTGGCTAAACCAAATGCCAAACAGCTTTTCCTTAGTGTTTGTTTTTCTTAAACAAGTTCAAACTCTTATCATATTGCTCCCCTCCCTTTGAAAATATGTACTCTGCAGATGCCTGAAATAACACACTCTCAGCAAATTGCAGCATTGGAAAAGTGAAGGTTATTTCATTCAGTCTAAACTCAATGGAAATTTTGTGGCTTAACAAGGAAATACCAAATACTTTATAAACTCCTTTAGAGCAGTAACCATGTCATATGACTCTGGTATCTCCTACCTACTAGGCTCATTGTCTACCTTCTCTGATGTCGTAATTTTAATAAGTAACTTGAATTGTTAACAATTTACTCTTGTCTTTTTCTTGGACTCAGAAATGTTATTTTATTATTATGTCTCTGCATCTTTCATGTGAGGTCCTTTGCAAACAGTTTTCTCCTAACTTGTTTTCATTGTGCAGATCATGTTGCAGGGTTTTCACATAACAGCTGAAAGGAATTAATAGGAGTTAAAGAAGCTTGCTTCAATTACAAGTAATTAATTTAGGCTTCACTTATCTCAAGGAATATCTGAATATACAAATTTACACATAAATCCTGGGAGTGTCAAATTATGCAACCTAGAGAAAGTAAGCTCTGCAGCTTTTTGGGCGTTGATTGCTATAGTCAGGGGTGTGTGTGTGGTTTTTGAATGAAATAGCAAAACCACTGCCAGTATTATCTACTTCACCTGGTTTTGAATAATTCCAGGAAGCTAGAAACTGAAAACCAGTCTTGTATAGAGTTAGAAAGATTGTAAATGCAGAAGAGGAAGAAAAAAACAAAACTAAACCATTTATCTATCCTCTTTGAACCCTTATAAGAGTTCACAATATTCCTCGTTTCAAATACTTGAAGTCTTCTCTGGGCAATGATTAATTTTTAAATAATTGTTTTTAATGTAAGACATAAAACATACGCACTGCAGATTTAAAGCATACTTGGAAGTAACAAGGAGAAACATAAAAATCGTTGATTCCACAGCATTTAATATATTGGTAACCTTCAATTTTTTTCTAGGCACGTTTTTATAAAATGAGCCAATACTATACAAACGTTTTTGTAACTTTTTTTAATTTGTACTATTTTGTGAGTACGTGAAAAAGAAATTTGTAAAGAGTAAACGATTACAGCATGTTGTGGTATTACACACTGTTGAAGGGCGCCATCTGTGGCGTGGTGGCTGCGCTTTGGTGGGAAGAGGTTCTCTTGGGGTTCCCGTTTTTCTCCCAGCTCTGGGTGGGCAATACTGACCACTCTCCGACCTAAAGGGGCTTCTCCGTTTGCCTTTGACGACCTGAGCTTAGGAGAATGGAGACACGTGGGAGTTCAGAGGAGGGCGTTCAGGGTGACTGCGAACACGAGCTGCCGGAGCTGTCCACATCTCCCCTAGGCAGGGCCCACTGGCCCCAGCCCGGGAAGGGAGCAAGGGCGGGAGGGCAGTTGGCAGTGGCACGGTCTGGGTCCAAATCTTTAGCCCCCCCCACCCCGGCTGGCGCCATTCTCGGGCGGTAAAGTGAGATAAAAGCAGGGCAAGGTTCTGCAACTCCAAATCAGGGAGGCGCAGCTCCTACACCAACGCCTTTCCGGGGCTCCGGGTGTGTTTGTTCCAACTGTTTAAACTGTTTCAAAGCGTCCGAACTCCAGCGACCTTCGCAAACAACTCTTTATCTCGCGGGCGAGAGCGCTGCCCTTATTTGCGGGGGAGGGCAAACTGAACGCCGGCACCGGGGAGCTAACGGAGACCTCCTCTAGGTCCCCCGCCTGCTGGGACCCCAGCTGGCAGTCCCTTCCCGCCCCCGGACCGCGAGCTTCTTGCGTCAGCCCCGGCGCGGGTGGGGGATTTTCGGAAGCTCAGCCCGCGCGGCCGGCGGGGGAAGGAAGGGCCCGGACTATTTTGTGAGTACGTGAAAAAGAAATTTGTAAAGAGTAAACGATTACAGCATGTTGTGGTATTACACACTGTTGAAGGGCGCCATCTGTGGCGTGGTGGCTGCGCTTTGGTGGGAAGAGGTTCTCTTGGGGTTCCCGTTTTTCTCCCAGCTCTGGGTGGGCAATACTGACCACTCTCCGACCTAAAGGGGCTTCTCCGTTTGCCTTTGACGACCTGAGCTTAGGAGAATGGAGACACGTGGGAGTTCAGAGGAGGGCGTTCAGGGTGACTGCGAACACGAGCTGCCGGAGCTGTCCACATCTCCCCTAGGCAGGGCCCACTGGCCCCAGCCCGGGAAGGGAGCAAGGGCGGGAGGGCAGTTGGCAGTGGCACGGTCTGGGTCCAAATCTTTAGCCCCCCCCACCCCGGCTGGCGCCATTCTCGGGCGGTAAAGTGAGATAAAAGCAGGGCAAGGTTCTGCAACTCCAAATCAGGGAGGCGCAGCTCCTACACCAACGCCTTTCCGGGGCTCCGGGTGTGTTTGTTCCAACTGTTTAAACTGTTTCAAAGCGTCCGAACTCCAGCGACCTTCGCAAACAACTCTTTATCTCGCGGGCGAGAGCGCTGCCCTTATTTGCGGGGGAGGGCAAACTGAACGCCGGCACCGGGGAGCTAACGGAGACCTCCTCTAGGTCCCCCGCCTGCTGGGACCCCAGCTGGCAGTCCCTTCCCGCCCCCGGACCGCGAGCTTCTTGCGTCAGCCCCGGCGCGGGTGGGGGATTTTCGGAAGCTCAGCCCGCGCGGCCGGCGGGGGAAGGAAGGGCCCGGACTCTTGCCCCGCCCTTGTGGGGCGGGAGGCGGAGCGGGGCAGGGGCCCGCCGGCGTGTAGCCGCGGCCGC |

**Supplementary Table 6**

| **Nrf2 mRNA 3’UTR sequence (Dual-luciferase reporter)** |
| --- |
| **WT:** ATTTAGGAGGATTTGACCTTTTCTGAGCTAGTTTTTTTGTACTATTATACTAAAAGCTCCTACTGTGATGTGAAATGCTCATACTTTATAAGTAATTCTATGCAAAATCATAGCCAAAACTAGTATAGAAAATAATACGAAACTTTAAAAAGCATTGGAGTGTCAGTATGTTGAATCAGTAGTTTCACTTTAACTGTAAACAATTTCTTAGG**A**CACCATTTGGGCTAGTTTCTGTGTAAGTGTAAATACTACAAAAACTTATTTATACTGTTCTTATGTCATTTGTTATATTCATAGATTTATATGATGATATGACATCTGGCTAAAAAGAAATTATTGCAAAACTAACCACTATGTACTTTTTTATAAATACTGTATGG**A**CAAAAAATGGCATTTTTTATATTAAATTGTTTAGCTCTGGCAAAAAAAAAAAATTTTAAGAGCTGGTACTAATAAAGGATTATTATGACTGTTAAA  **Mut:**  ATTTAGGAGGATTTGACCTTTTCTGAGCTAGTTTTTTTGTACTATTATACTAAAAGCTCCTACTGTGATGTGAAATGCTCATACTTTATAAGTAATTCTATGCAAAATCATAGCCAAAACTAGTATAGAAAATAATACGAAACTTTAAAAAGCATTGGAGTGTCAGTATGTTGAATCAGTAGTTTCACTTTAACTGTAAACAATTTCTTAGG**C**CACCATTTGGGCTAGTTTCTGTGTAAGTGTAAATACTACAAAAACTTATTTATACTGTTCTTATGTCATTTGTTATATTCATAGATTTATATGATGATATGACATCTGGCTAAAAAGAAATTATTGCAAAACTAACCACTATGTACTTTTTTATAAATACTGTATGG**C**CAAAAAATGGCATTTTTTATATTAAATTGTTTAGCTCTGGCAAAAAAAAAAAATTTTAAGAGCTGGTACTAATAAAGGATTATTATGACTGTTAAA |
