# Supplementary figures and images for: Epigenetically silenced lncRNA SNAI3-AS1 promotes ferroptosis in glioma via perturbing the m6A-dependent recognition of Nrf2 mRNA mediated by SND1
Source: J Exp Clin Cancer Res. 2023 May 19;42:127. doi: 10.1186/s13046-023-02684-3 (PMC10197824; doi:10.1186/s13046-023-02684-3)

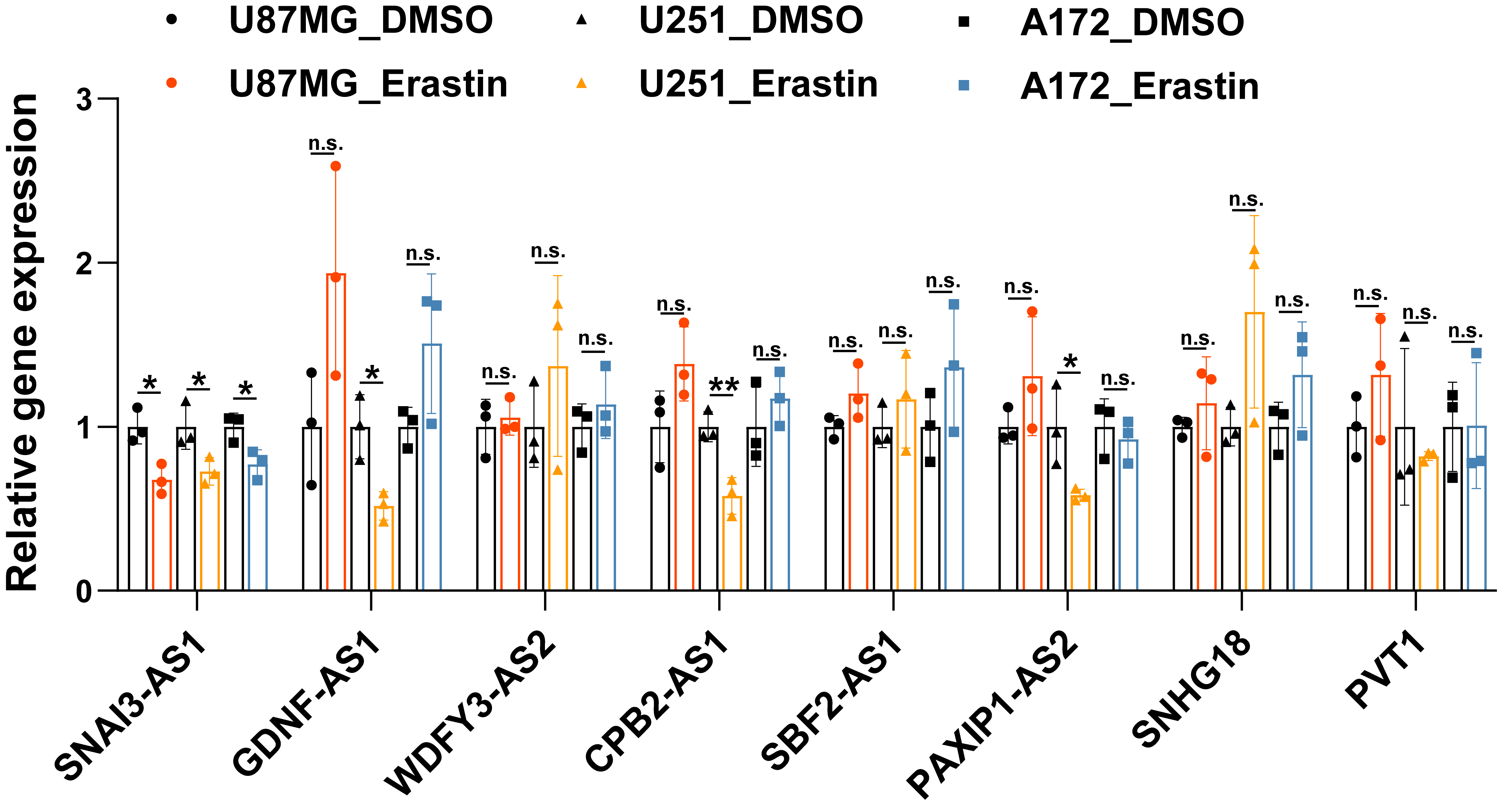

Supplement: Supplementary file 4 — Additional file 4: Supplementary Fig. 1. The expression of eight candidate lncRNAs in U87MG, U251 and A172 cells under erastin (5 μM, 48 h) treatments as measured by RT qPCR. *P < 0.05, and n.s., not significant. [file 13046_2023_2684_MOESM4_ESM.tif]

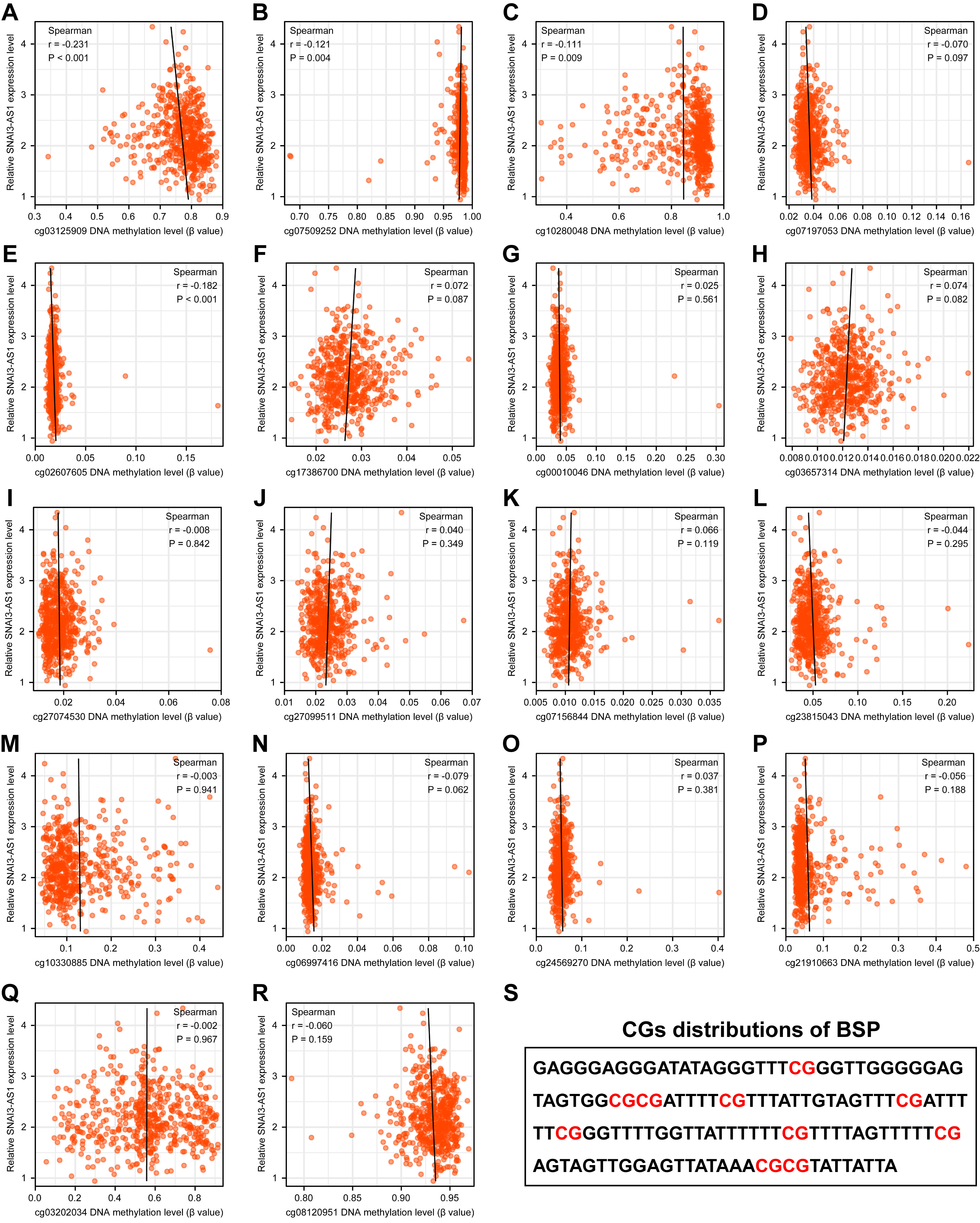

Supplement: Supplementary file 5 — Additional file 5: Supplementary Fig. 2. (A-R) Correlation between SNAI3-AS1 expression and 18 CpG sites of SNAI3-AS1 DNA promoter. (S) CG islands distribution of BSP. [file 13046_2023_2684_MOESM5_ESM.tif]

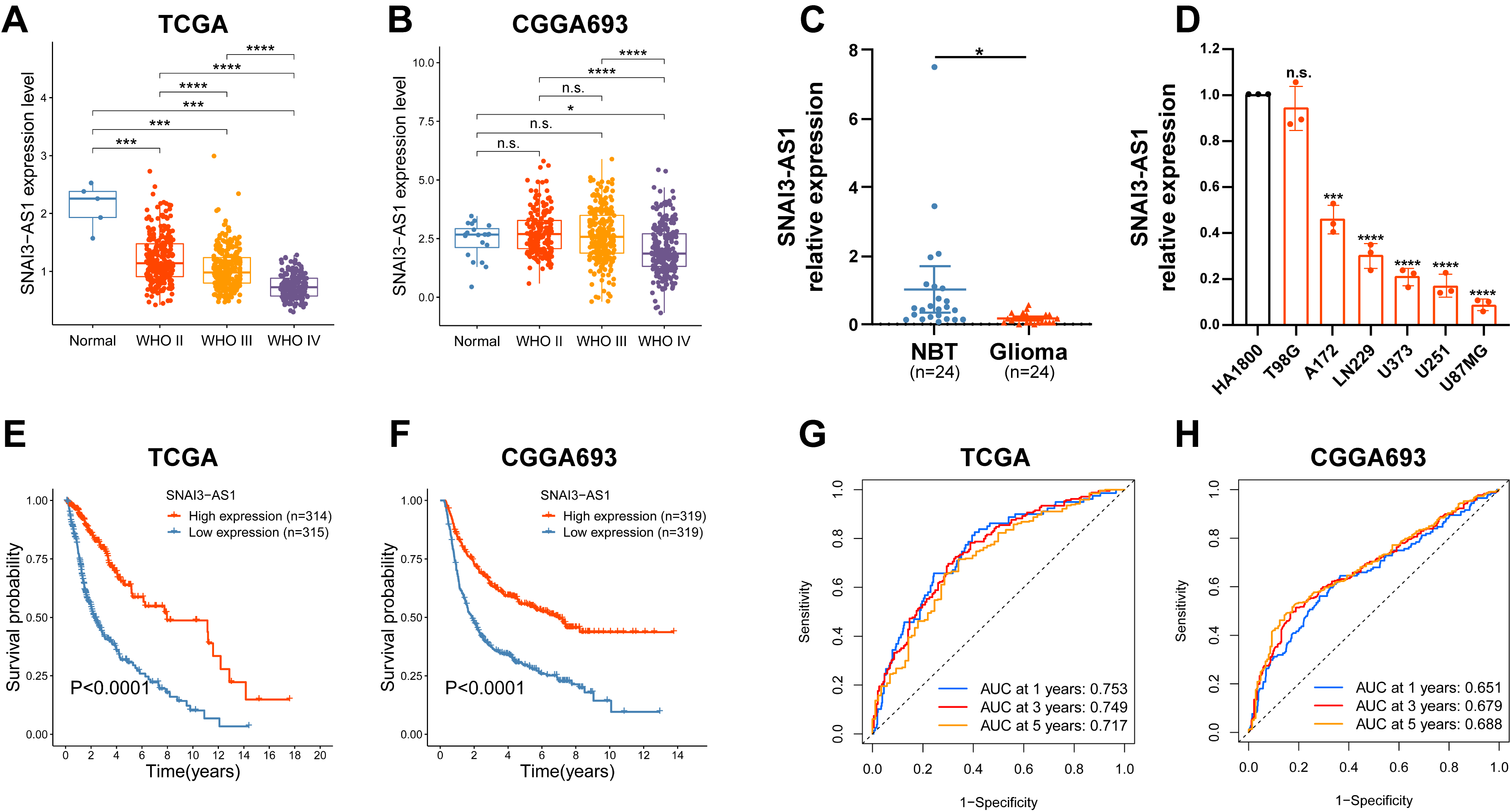

Supplement: Supplementary file 6 — Additional file 6: Supplementary Fig. 3. (A-B) Comparing the expression of SNAI3-AS1 between normal tissues and different WHO grade tissues based on TCGA and CGGA693databases. (C) SNAI3-AS1 expression was determined in 24 paired glioma tissues and nontumoral brain tissues via RT-qPCR. NBT, nontumoral brain tissue. (D) SNAI3-AS1 expression was determined in normal astrocyte and several glioma cell lines via RT-qPCR. (E-F) Kaplan–Meier analysis based on SNAI3-AS1 expression in glioma using the data from TCGA and CGGA693 databases. (G-H) The receiver operating characteristic (ROC) curves of SNAI3-AS1 in glioma using the data from TCGA and CGGA693 databases. *P < 0.05, ***P < 0.001, ****P < 0.0001, and n.s., not significant. [file 13046_2023_2684_MOESM6_ESM.tif]

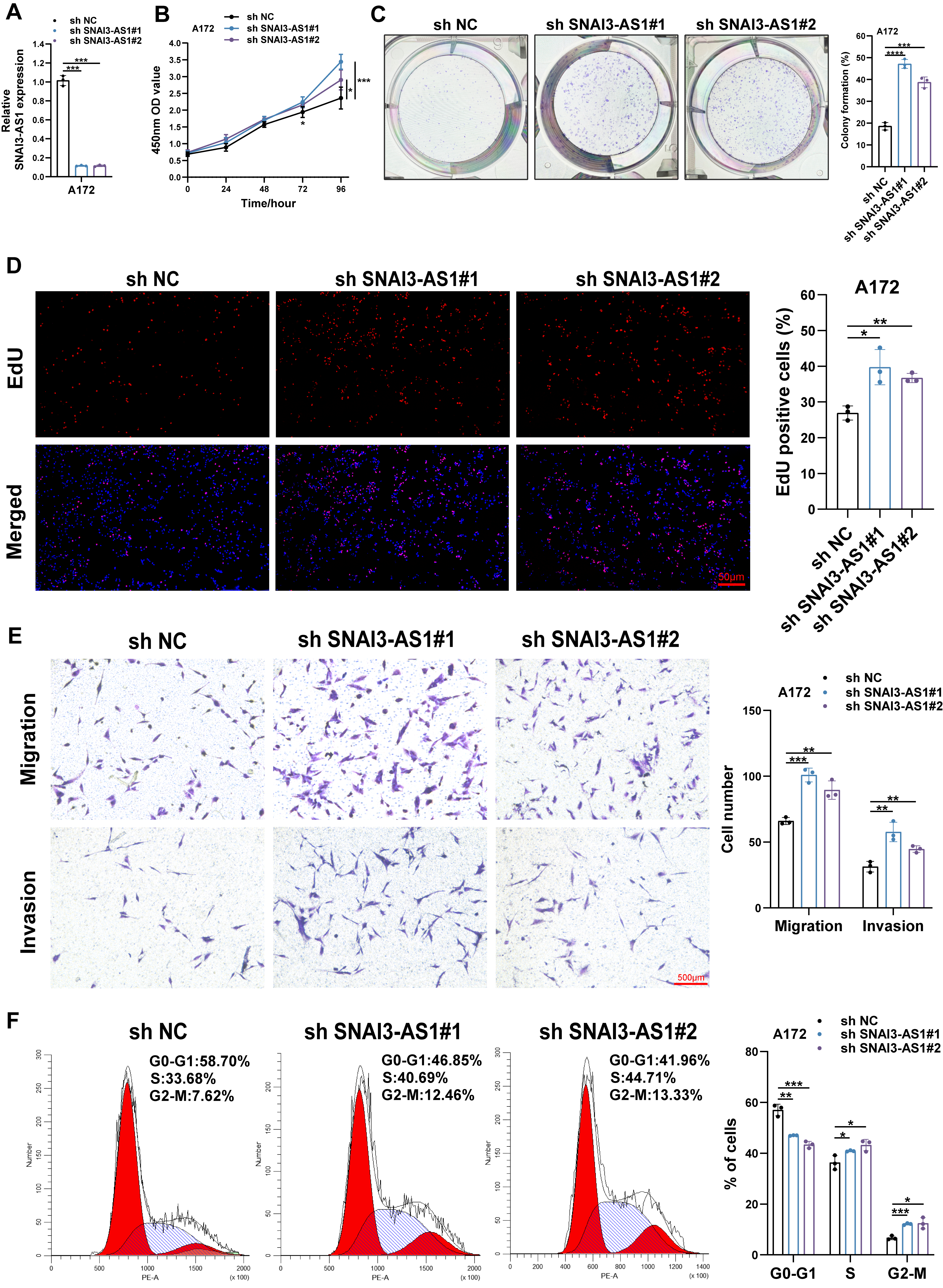

Supplement: Supplementary file 7 — Additional file 7: Supplementary Fig. 4. (A) RT-qPCR was used to detect the expression of SNAI3-AS1 in A172 cells transfected with two sh SNAI3-AS1 lentiviral vectors or control vector. (B) The growth curves of transfected A172 cells were determined by CCK8 assays. (C) The colony formation assays were performed in transfected A172 cells. (D) The proliferation of transfected A172 cells was detected by EdU staining assays. (E) The transwell assays showed the migration and invasion abilities of transfected A172 cells. (F) Cell cycle distributions of transfected A172 cells were measured by flow cytometry. *P < 0.05, **P < 0.01, ***P < 0.001, and ****P < 0.0001. [file 13046_2023_2684_MOESM7_ESM.tif]

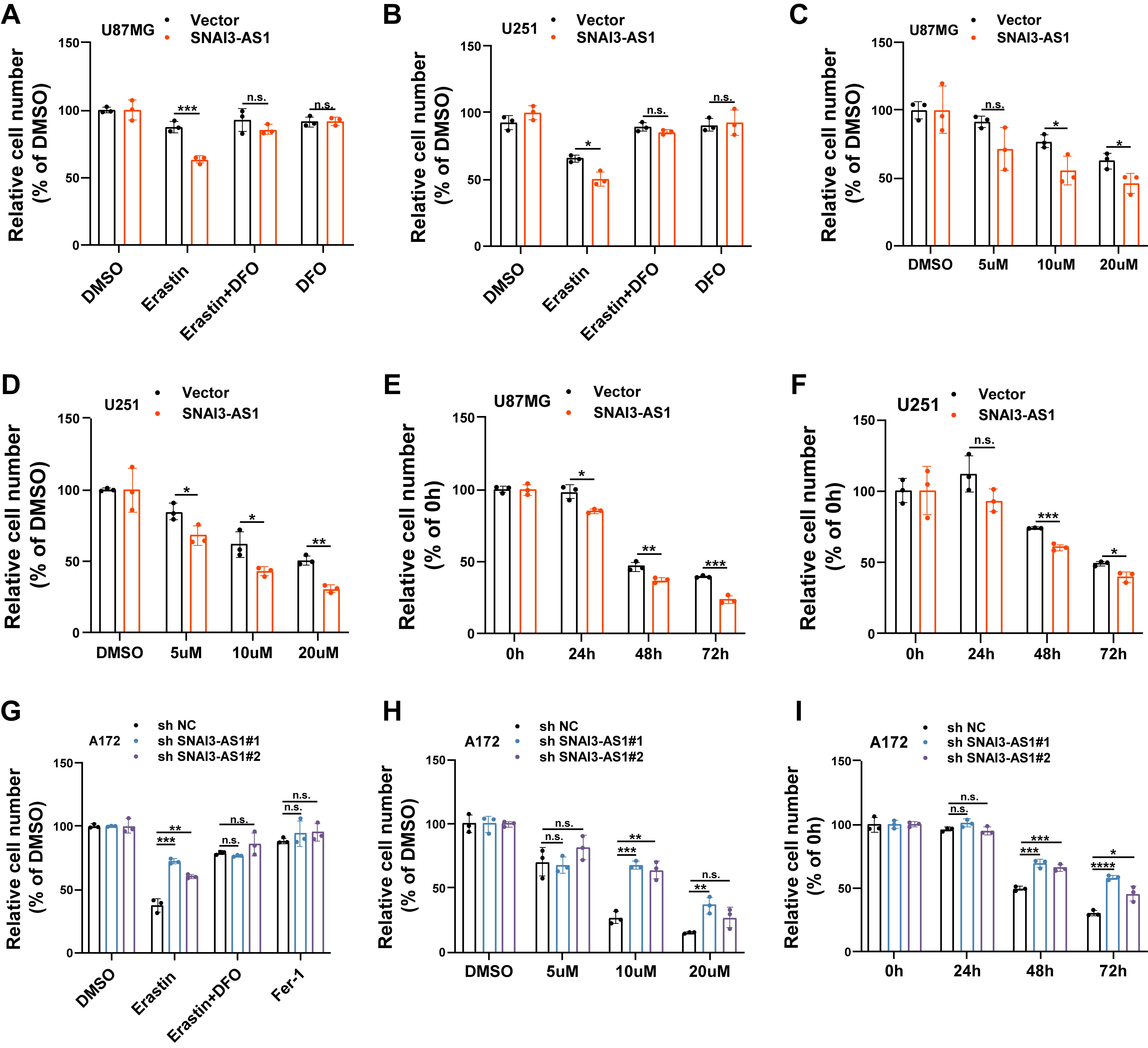

Supplement: Supplementary file 8 — Additional file 8: Supplementary Fig. 5. (A-F) U87MG and U251 cells stably overexpressing SNAI3-AS1 were treated with erastin (10 μM) ± Deferoxamine (DFO, 100 μM) for 48 h, different concentration of erastin (5/10/20 µM) for 48 h, or 10 µM erastin for 24/48/72 h. Cell viabilities were detected via CCK8 assays. (G-I) A172 cells with stable SNAI3-AS1 knockdown were treated with erastin (10 μM) ± Deferoxamine (DFO, 100 μM) for 48 h, different concentration of erastin (5/10/20 µM) for 48 h, or 10 µM erastin for 24/48/72 h. Cell viabilities were detected via CCK8 assays. *P < 0.05, **P < 0.01, ***P < 0.001, ****P < 0.0001, and n.s., not significant. [file 13046_2023_2684_MOESM8_ESM.tif]

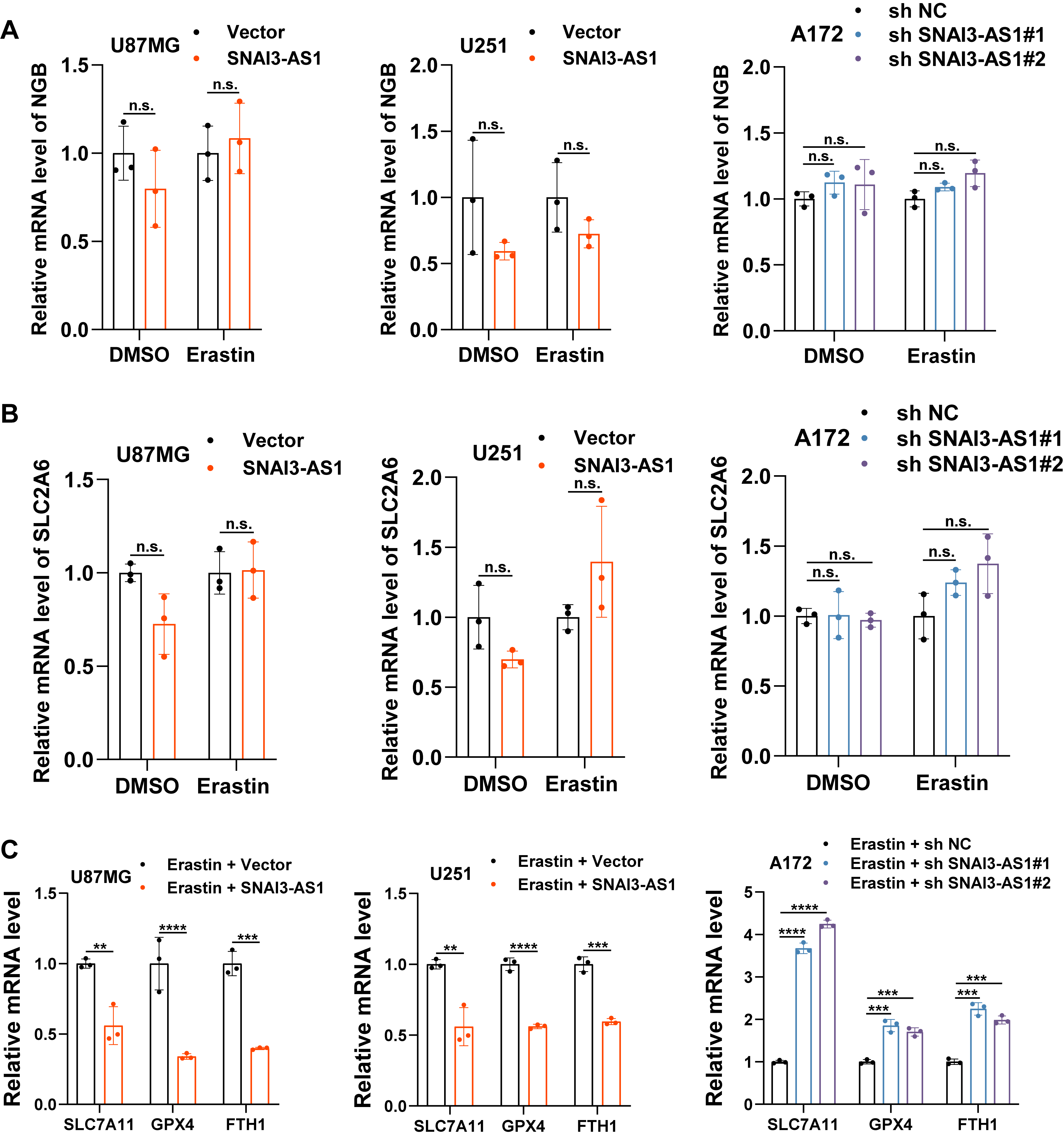

Supplement: Supplementary file 9 — Additional file 9: Supplementary Fig. 6. RT-qPCR was used to detect the mRNA levels of NGB (A) and SLC2A6 (B) after SNAI3-AS1 overexpression or knockdown under DMSO and erastin (10 μM, 48 h) treatments. (C) The mRNA levels of SLC7A11, GPX4, and FTH1 were measured in glioma cells with SNAI3-AS1 overexpression or knockdown and erastin (10 μM, 48 h) treatments. **P < 0.01, ***P < 0.001, ****P < 0.0001, and n.s., not significant. [file 13046_2023_2684_MOESM9_ESM.tif]

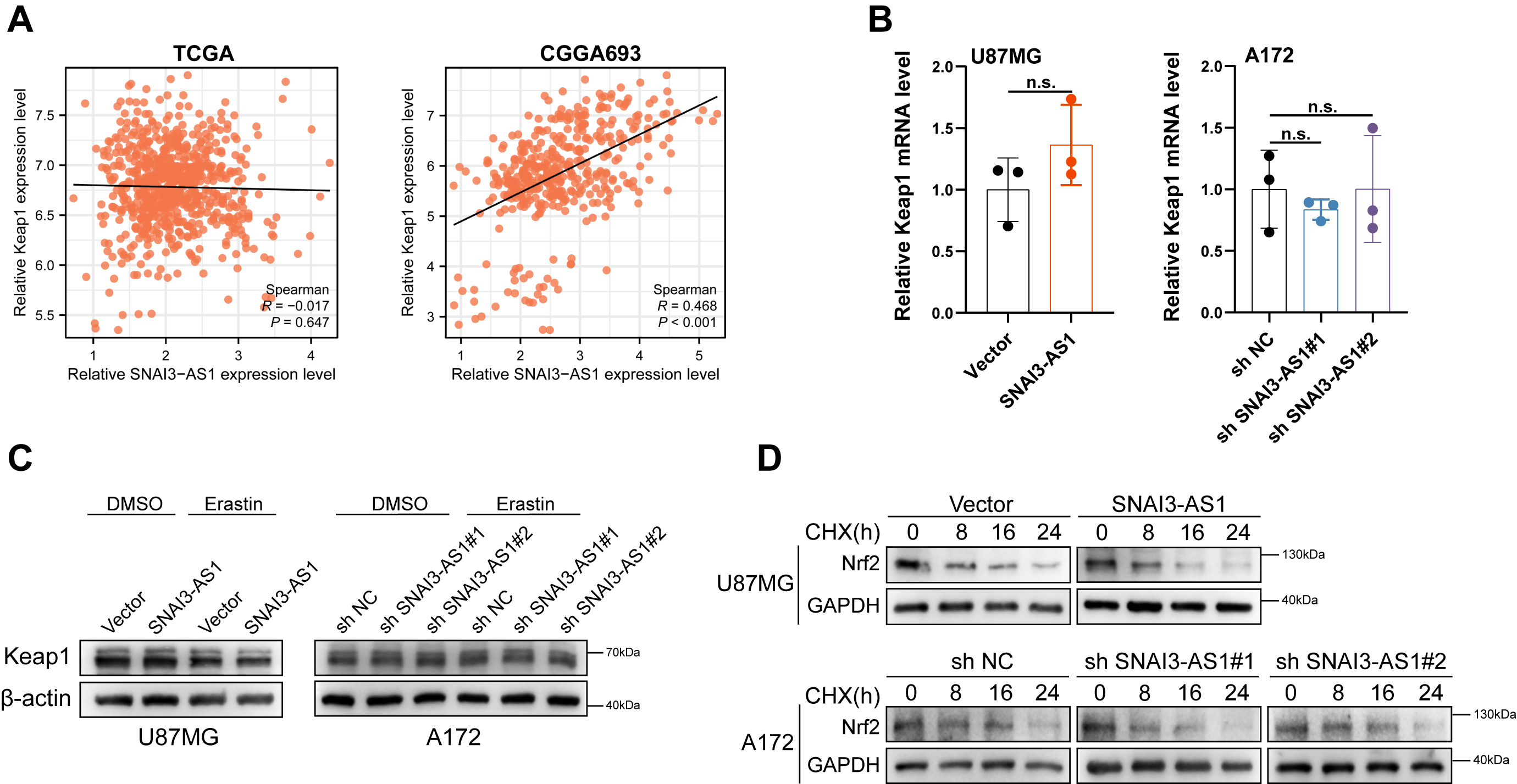

Supplement: Supplementary file 10 — Additional file 10: Supplementary Fig. 7. (A) The correlations between SNAI3-AS1 and Keap1 in TCGA and CGGA693 databases. (B) The mRNA level of Keap1 detected by RT-qPCR after SNAI3-AS1 overexpression or knockdown. (C) The protein level of Keap1 detected by WB after SNAI3-AS1 overexpression or knockdown. (D) The Nrf2 protein level in indicated time point after treated with cycloheximide (CHX, 10 µg/ mL) in glioma cells with SNAI3-AS1 overexpression or knockdown. n.s., not significant. [file 13046_2023_2684_MOESM10_ESM.tif]

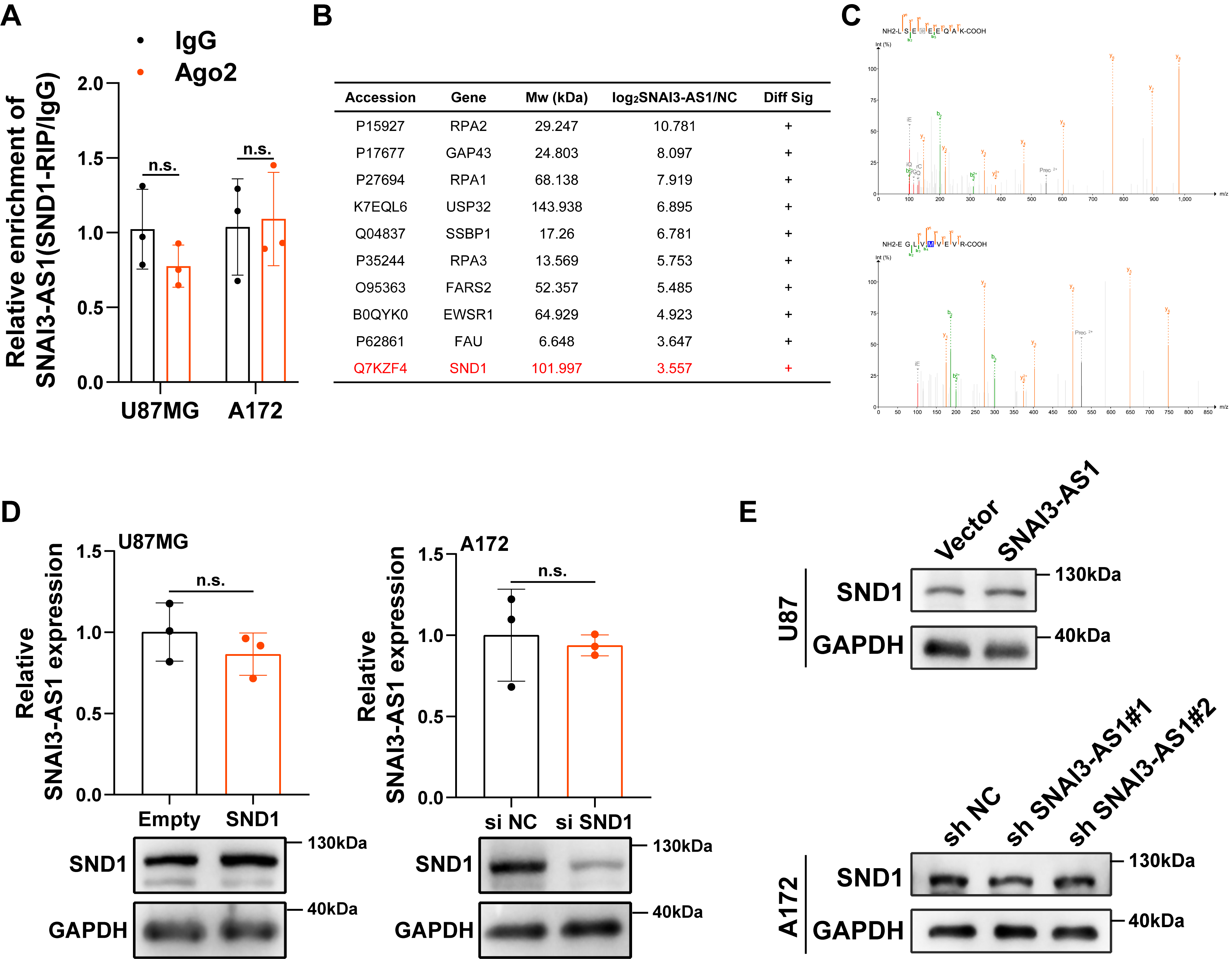

Supplement: Supplementary file 11 — Additional file 11: Supplementary Fig. 8. (A) RIP assays were performed using anti-Ago2 and IgG antibodies. The enrichments of SNAI3-AS1 by Ago2 or IgG were detected via RT-qPCR. (B) Top 10 possible candidate binding proteins of SNAI3-AS1 based on the mass spectrometry analysis. (C) Mass spectrometry revealed SND1 peptides pulled down by SNAI3-AS1 probe. (D) The expression of SNAI3-AS1 detected by RT-qPCR after overexpressing SND1 or silencing SND1. (E) Western blotting showed the expression of SND1 protein after SNAI3-AS1 overexpression or knockdown. n.s., not significant. [file 13046_2023_2684_MOESM11_ESM.tif]

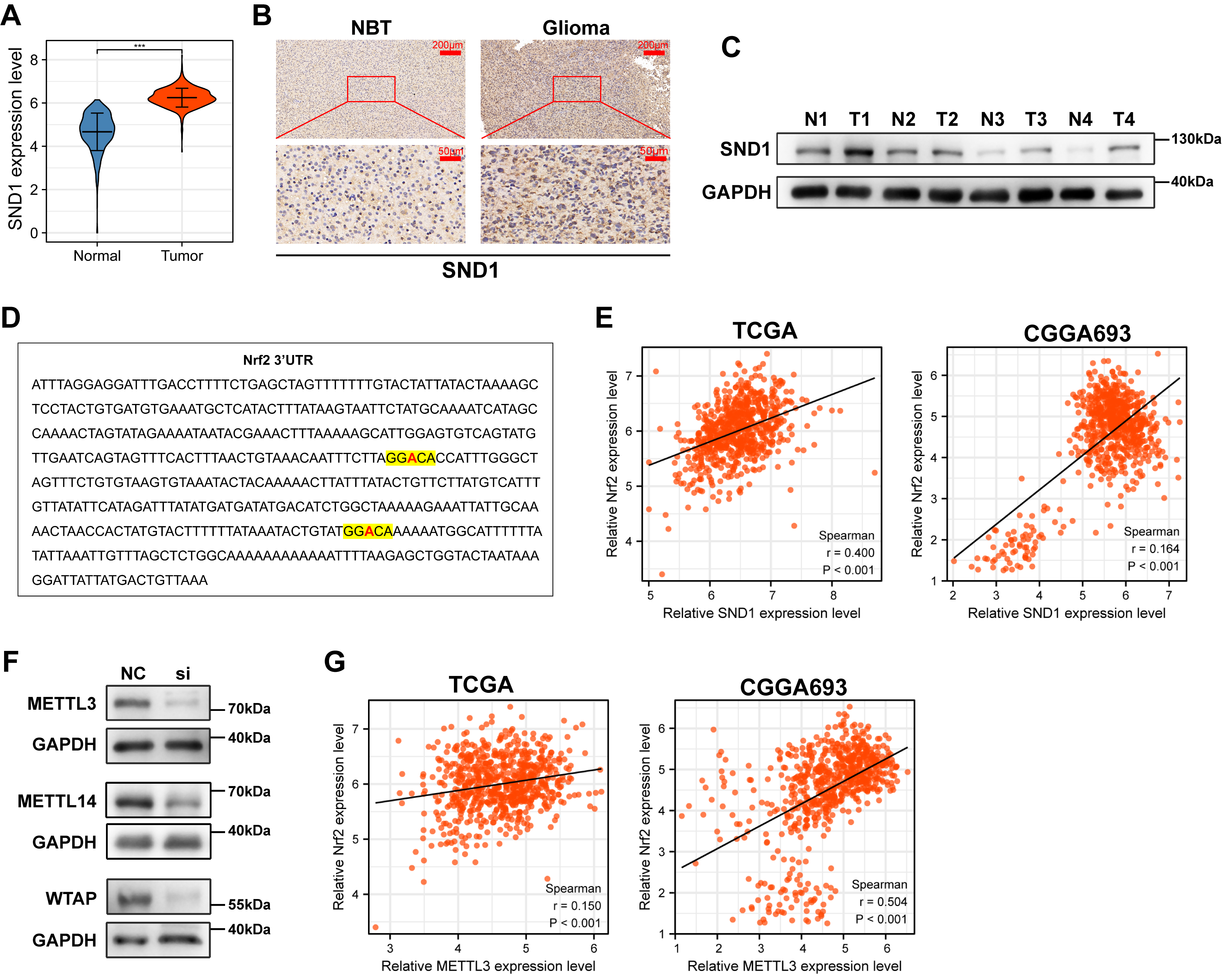

Supplement: Supplementary file 12 — Additional file 12: Supplementary Fig. 9. (A) Comparing the expression of SND1 between normal tissues from GTEx database and glioma tissues from TCGA database. (B) SND1 expressions in a paired glioma tissue and nontumoral brain tissue showed by IHC. (C) SND1 expressions in 4 paired glioma tissue and nontumoral brain tissue showed by western blotting. (D) The sequences of Nrf2 mRNA 3’UTR. (E) The correlation between SND1 and Nrf2 in TCGA and CGGA693 databases. (F) Silencing METTL3, METTL14, and WTAP in U87MG cells were verified by western blotting. (G) The correlation between METTL3 and Nrf2 in TCGA and CGGA693 databases. [file 13046_2023_2684_MOESM12_ESM.tif]
